# The Cost Effectiveness of Psychological and Pharmacological Interventions for Social Anxiety Disorder: A Model-Based Economic Analysis

**DOI:** 10.1371/journal.pone.0140704

**Published:** 2015-10-27

**Authors:** Ifigeneia Mavranezouli, Evan Mayo-Wilson, Sofia Dias, Kayleigh Kew, David M. Clark, A. E. Ades, Stephen Pilling

**Affiliations:** 1 National Collaborating Centre for Mental Health, Centre for Outcomes Research and Effectiveness, Research Department of Clinical, Educational & Health Psychology, University College London, London, United Kingdom; 2 School of Social and Community Medicine, University of Bristol, Bristol, United Kingdom; 3 Department of Experimental Psychology, University of Oxford & Oxford Cognitive Health NIHR Clinical Research Facility, Oxford, United Kingdom; Deakin University, AUSTRALIA

## Abstract

**Background:**

Social anxiety disorder is one of the most persistent and common anxiety disorders. Individually delivered psychological therapies are the most effective treatment options for adults with social anxiety disorder, but they are associated with high intervention costs. Therefore, the objective of this study was to assess the relative cost effectiveness of a variety of psychological and pharmacological interventions for adults with social anxiety disorder.

**Methods:**

A decision-analytic model was constructed to compare costs and quality adjusted life years (QALYs) of 28 interventions for social anxiety disorder from the perspective of the British National Health Service and personal social services. Efficacy data were derived from a systematic review and network meta-analysis. Other model input parameters were based on published literature and national sources, supplemented by expert opinion.

**Results:**

Individual cognitive therapy was the most cost-effective intervention for adults with social anxiety disorder, followed by generic individual cognitive behavioural therapy (CBT), phenelzine and book-based self-help without support. Other drugs, group-based psychological interventions and other individually delivered psychological interventions were less cost-effective. Results were influenced by limited evidence suggesting superiority of psychological interventions over drugs in retaining long-term effects. The analysis did not take into account side effects of drugs.

**Conclusion:**

Various forms of individually delivered CBT appear to be the most cost-effective options for the treatment of adults with social anxiety disorder. Consideration of side effects of drugs would only strengthen this conclusion, as it would improve even further the cost effectiveness of individually delivered CBT relative to phenelzine, which was the next most cost-effective option, due to the serious side effects associated with phenelzine. Further research needs to determine more accurately the long-term comparative benefits and harms of psychological and pharmacological interventions for social anxiety disorder and establish their relative cost effectiveness with greater certainty.

## Introduction

Social anxiety disorder is one of the most persistent and common anxiety disorders, with a lifetime prevalence estimated to range between 3.9% and 13.7% in Europe [1]. People with social anxiety disorder have difficulty forming and retaining personal and social relationships [2], have higher risk of leaving school early and obtaining poorer qualifications [3], experience impairment in their daily functioning including work/school performance and social life [4], and report an important reduction in their quality of life compared with people without the disorder [5]. They also incur considerable healthcare costs, especially relating to the use of primary care services, experience high levels of productivity losses and receive higher social benefits compared with people in the general population [6–8]. It has been shown that as the number of social fears increases, so does health service utilisation [9]. The presence of comorbid psychiatric disorders increases usage of health services and productivity losses [6,8,9].

Several studies have assessed the clinical effectiveness of psychological and pharmacological treatments for social anxiety disorder [10–13]. Recently, individually delivered psychological therapies were demonstrated to be more effective than drugs and self-help for adults with social anxiety disorder [14]. Given the variety of available interventions for the treatment of social anxiety disorder, the high costs associated with provision of psychological interventions, and the need for efficient use of healthcare resources under conditions of restricted budgets, the objective of this study was to examine the cost-effectiveness of a wide range of psychological and pharmacological interventions for the treatment of adults with social anxiety disorder from the perspective of the British National Health Service (NHS) and Personal Social Services, using decision-analytic modelling.

This study is an update of the economic analysis that informed the development of national clinical guidance for social anxiety disorder in England and Wales, published by the National Institute for Health and Care Excellence (NICE) [15]. The guideline was developed by the Guideline Development Group (GDG), a multi-disciplinary team consisting of clinical academics, health professionals and service user and carer representatives with expertise and experience in the field of social anxiety. The GDG contributed to the development of the economic model by providing advice on issues relating to the natural history and treatment patterns of social anxiety disorder in the UK, as well as on model inputs in areas where evidence was lacking. The analysis presented here utilised efficacy data from an updated systematic review and network meta-analysis (NMA) of interventions for adults with social anxiety disorder [14].

## Methods

### Study population

The study population comprised adults aged at least 18 years who fulfilled diagnostic criteria for social anxiety disorder and were eligible for all the treatment options included in the analysis. Full details on the study population selection criteria are provided in a related publication [14].

### Interventions assessed

The economic analysis assessed interventions that the GDG considered as appropriate first-line treatments for adults with social anxiety disorder, for which adequate clinical evidence was available. The interventions evaluated in the economic analysis were selected among those included in a recent comprehensive systematic review and NMA [14]. We evaluated the cost effectiveness of distinct interventions, rather than classes of treatments, as there may be differences in resource implications among interventions belonging to the same class. Details on the rationale for the selection of appropriate interventions for consideration in the economic analysis are reported in the NICE full guideline report [16]. The following interventions were considered:

Pharmacological interventions:

- Seven selective serotonin / serotonin-noradrenergic reuptake inhibitors (SSRIs / SNRIs): citalopram, escitalopram, fluoxetine, fluvoxamine, paroxetine, sertraline and venlafaxine (prolonged-release formulation XL)
- Two monoamine oxidase inhibitors (MAOIs): moclobemide and phenelzine
- One anticonvulsant: pregabalin
- One noradrenergic and specific serotonergic antidepressant: mirtazapine

Psychological interventions:

- Self-help without therapist support: book-based (SHNS, book) and computer-based (SHNS, internet), both involving minimal contact with therapists
- Self-help with therapist support: book-based (SHWS, book) and computer-based (SHWS, internet)
- Exposure in vivo
- Mindfulness training
- Two types of group cognitive behavioural therapy: group cognitive behavioural therapy following the Heimberg model (GCBT Heimberg) [17]; and group cognitive behavioural therapy not following a specified model (GCBT general)
- Interpersonal psychotherapy (IPT)
- Psychodynamic psychotherapy (PDPT)
- Supportive therapy
- Four types of individually delivered cognitive behavioural therapy: individual cognitive behavioural therapy following the Hope, Heimberg and Turk model (ICBT Hope) [18]; cognitive therapy following the Clark and Wells model (ICBT C&W) [19]; cognitive therapy following the Clark and Wells model with shortened sessions (ICBT short); and individual cognitive behavioural therapy not following a specified model (ICBT general)

The economic model also included two ‘inactive’ interventions, pill placebo and wait list, in order to assess the cost effectiveness of active interventions versus non-specific medical management and a do-nothing option, respectively.

The economic analysis made simultaneous comparisons of all the above treatment options; this was enabled by employing NMA methods to synthesise available clinical evidence [20].

### Economic model structure

**Fig 1:**
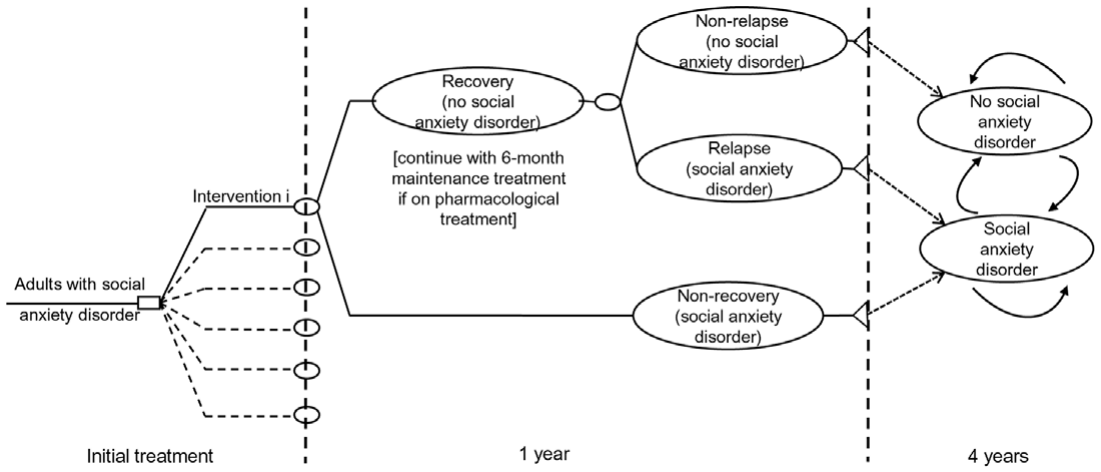
Schematic diagram of the economic model constructed to assess the cost-effectiveness of interventions for social anxiety disorder.

A hybrid decision-analytic model consisting of a decision-tree followed by a two-state Markov model was constructed using Microsoft Office Excel 2007 to assess total costs and Quality Adjusted Life Years (QALYs) of the 28 interventions for social anxiety disorder. The model structure, shown in Fig 1, was dictated by the natural history of the disorder, its treatment patterns and associated care pathways in the UK, and the availability of relevant clinical and epidemiological data.

The model followed hypothetical cohorts of adults with social anxiety disorder initiated on each of the 28 interventions assessed. For purposes of estimation of QALYs, initial treatment lasted 12 weeks for all interventions, although in trials and clinical practice the duration of psychological interventions ranges between 9 and 16 weeks; this modelling assumption did not affect estimated resource use. Following treatment, people in each cohort either recovered, no further meeting criteria for diagnosis (thus entering a state of ‘no social anxiety disorder’) or failed to recover (remaining in the state of ‘social anxiety disorder’). People who recoved following pharmacological treatment were assumed to receive another 26 weeks of maintenace treatment with the same drug; people recovering after psychological treatment did not receive further maintenance treatment. Those not recovering were assumed to stop treatment rather than switch to an alternative intervention as the GDG suggested this is likely in a population that is usually reluctant to maintain contact with health services.

During the year post-treatment, people who did not recover remained in the ‘social anxiety disorder’ state. Those who recovered might relapse, meeting again diagnostic criteria for the disorder, and thus re-enter the ‘social anxiety disorder’ state. From that point on all people in each cohort entered the Markov model, which was run in yearly cycles. During each cycle people remained in their current health state or moved between the ‘no social anxiety disorder’ and the ‘social anxiety disorder’ states. A half-cycle correction was applied. The yearly cycles were considered appropriate and consistent with the slow, chronic course of social anxiety disorder and the low rates of clinical changes in terms of recovery and relapse characterising the disorder [21], and were selected for convenience in terms of applying annual discounting of costs and benefits.

The time horizon of the analysis was the 12 weeks of initial treatment plus 5 years post-treatment, comprising 1 year in the decision-tree and 4 yearly cycles in the Markov model. This time horizon was selected to enable assessment of longer term benefits and costs associated with each intervention and to allow intervention costs to be spread over a longer time period, over which benefits of interventions can be still potentially enjoyed. A 5-year time horizon following 12 weeks of initial treatment was considered appropriate and reasonable, given that long-term clinical data were available from a prospective naturalistic study that observed the long-term clinical course of anxiety disorders including social anxiety disorder over 12 years [21], so there was no need for extrapolation of clinical data.

### Clinical data utilised in the model

#### Clinical efficacy

Efficacy data were derived from a systematic literature review and NMA of randomised controlled trials (RCTs) of interventions for adults with social anxiety disorder [14]; the NMA, based on a random effects model [22], was conducted within a Bayesian framework using Markov Chain Monte Carlo simulation techniques implemented in WinBUGS 1.4 [23,24]. The NMA included 101 trials reporting continuous data on symptom scales of social anxiety, of which 24 also reported dichotomous recovery data (with recovery being defined as no longer meeting criteria for diagnosis). The studies reported several continuous measures of social anxiety, none of which were common to all trials, so treatment effects for each trial were calculated as a standardised mean difference (SMD). Based on published psychometric properties and data from clinically referred participants who completed several measures, continuous measures were assumed to be equally responsive and had a mean correlation of 0.65 (for details see [14]). The outcome measures used in the clinical analysis are provided in S1 File.

The log-odds ratio of recovery was transformed into a notional SMD for recovery using the formula $LOR_{\text{Recovery}}=-\frac{\pi }{\sqrt{3}}SMD$ [25].

Following inspection of the relationship between the log-odds ratio estimated from the SMD of continuous data and the transformed log-odds ratio of recovery data in studies reporting both ([14]—see Appendix 4 in Supplementary Appendix A), we assumed a linear relationship between the pooled treatment effects of symptom scales and the transformed treatment effects on recovery that was the same across all treatments, and forced the intercept at zero. The plausibility of the assumed relationship between the log-odds ratio of the SMD of continuous data and the transformed log-odds ratio of recovery data was assessed empirically by examining the relationship in the relative effects for studies reporting both outcomes, and discussed with clinical experts who considered it reasonable.

Thus, relative treatment effects expressed as SMD versus wait list were estimated from all studies and this correspondence (between the log-odds ratio of SMD of continuous data and the transformed log-odds ratio of recovery data) and its uncertainty were used to borrow strength from studies that reported recovery, and also to transform the estimates into log-odds ratios of recovery in order to populate the economic model. These were subsequently used to estimate absolute probabilities of recovery for each intervention using the probability of recovery for wait list as baseline, which was calculated by pooling recovery data from all wait list arms in RCTs included in the NMA. We note that the method used fully incorporates the uncertainty in the estimates.

The WinBUGS model was run with an initial burn-in period of 20,000 iterations, followed by 40,000 further iterations, thinned by 4 so as to obtain 10,000 iterations for use in the economic model. Full details of the methods and results of the systematic literature review and NMA, including risk of bias assessment of the individual studies, the full dataset, the statistical analysis of clinical data and the statistical code used in the NMA are provided in a related publication [14].

#### Other clinical input parameters

The first-year probability of relapse after recovery with a drug was estimated using pooled data from 5 placebo-controlled pharmacological RCTs on relapse prevention in adults with social anxiety disorder [26–30], identified by a systematic literature search; this probability was estimated to be higher that the pooled risk of relapse *during* maintenance treatment in RCT active drug arms, but lower than the pooled risk of relapse of responders to initial drug treatment who were subsequently randomised to placebo (thus not receiving maintenance treatment); for simplicity and due to lack of more suitable data the first year probability of relapse for drugs was assumed to equal the midpoint of the two pooled risks. The resulting estimate, which was validated by GDG expert opinion, was utilised across all pharmacological treatment arms of the model and pill placebo, as drug-specific relapse data were sparse and mostly unavailable.

The first-year probability of relapse after recovery with psychological interventions was calculated by dividing the first-year probability of relapse for drugs by the risk ratio of relapse of drugs versus psychological therapies. The latter was estimated from data derived from a small observational 12-month follow-up study [31] of responders in an RCT of phenelzine versus group CBT for social anxiety disorder [32] and was applied to all psychological interventions due to lack of intervention-specific relapse data.

The first-year probability of relapse after recovery in wait list was estimated using data from a naturalistic study that reported 12-year probabilities of recovery and recurrence in adults with social anxiety disorder estimated using survival analysis [21]. Data from this study were also used to calculate the annual probabilities of recovery and relapse in years 2 to 5 post-treatment (i.e. in the Markov component of the model), which were applied to all model cohorts, regardless of initial treatment.

### Utility data considered in the model

Utility scores express preferences for different states of health-related quality of life (HRQoL) and are necessary for the estimation of QALYs. Following a systematic literature search of utility data for social anxiety disorder, the economic model was populated with utility scores obtained from a Finish national health survey [33] that reported EQ-5D utility scores (estimated using the UK Time Trade-Off Tarrif) [34] for people with social anxiety disorder and people with no mental disorder over the last 12 months. The 12-month prevalence of psychiatric disorders in survey participants was assesed using the Munich version of the Composite International Diagnostic Interview (M-CIDI) [35]. Utility scores for people with social anxiety disorder over the previous 12 months were used for the ‘social anxiety disorder’ state. Utility scores for people with no mental disorder over the last 12 months were used as a proxy for the state of ‘no social anxiety disorder’, although it is acknowledged that people recovering from social anxiety disorder may not reach the HRQoL of a person without a mental disorder, and thus their HRQoL may have been overestimated, at least over the first few months following recovery.

Another limitation of using these data was that diagnosis referred to a 12-month period prior to data collection, so some survey participants might have experienced an improvement in their condition over this period (and actually might have recovered at the point of interview), and thus the HRQoL associated with the ‘social anxiety disorder’ state may have been overestimated. These limitations were deemed acceptable as no better quality utility data were identified; moreover, since overestimation of utility scores was likely to have occurred, to some extent, in both ‘social anxiety disorder’ and ‘no social anxiety disorder’ states, the effect of overestimation was deemed to be, at least partially, cancelled out and to be affecting the results of the economic analysis only insignificantly. The economic model assumed linear changes in utility when transitioning between ‘social anxiety disorder’ and ‘no social anxiety disorder’.

### Healthcare resource use and cost data

The analysis considered intervention costs and other health and personal social service costs associated with social anxiety disorder, expressed in 2015 prices.

Pharmacological intervention costs consisted of drug acquisition and general practitioner (GP) visit costs. The intervention cost of pill placebo comprised GP visit costs only. The average daily dosage for each drug was determined according to optimal clinical practice [36] and was consistent with dosages reported in the RCTs that were included in the NMA. Initial drug treatment lasted 12 weeks, followed, in people who recovered, by 26 weeks of maintenance treatment at the same daily dosage, according to optimal practice. Initial drug treatment included 4 GP visits; maintenance treatment for those recovering included 3 extra GP visits. These resource use estimates were based on the GDG expert opinion and are lower than descriptions of medical resource use in pharmacological trial protocols. Drug acquisition costs and the GP unit cost were taken from national sources [37,38]. Active drug intervention costs, including 7 GP visits, ranged from £339 (citalopram) to £939 (pregabalin) (Table 1).

**Table 1:** Intervention costs of pharmacological treatments considered in the economic analysis (2015 prices).

Drug	Mean daily dosage	Drug cost—12 weeks^a^	Drug cost—26 weeks^a^	Total intervention (drug and GP^b^) cost—12+26 weeks
Citalopram	40 mg	£3.84	£8.32	£339.43
Escitalopram	20 mg	£6.21	£13.46	£346.93
Fluoxetine	40 mg	£6.50	£14.07	£347.84
Fluvoxamine	150 mg	£71.48	£154.88	£553.64
Mirtazapine	30 mg	£4.80	£10.40	£342.47
Moclobemide	600 mg	£78.34	£169.75	£575.36
Paroxetine	40 mg	£12.82	£27.79	£367.88
Phenelzine	60 mg	£75.60	£163.80	£566.67
Pregabalin	450 mg	£193.20	£418.60	£939.07
Sertraline	200 mg	£9.96	£21.58	£358.81
Venlafaxine	150 mg	£52.36	£113.45	£493.08
Pill placebo	NA	NA	NA	£327.27

^a^ Drug acquisition costs were taken from the Electronic Drug Tariff for England and Wales [37]; lowest reported price for each drug was used, including prices of generic forms, where available.

^b^ GP cost includes 4 GP visits during 12 weeks of initial treatment and 3 visits during the 26-week maintenance period, at £46.75 per visit = £327.25; GP unit cost, including qualification and direct care staff costs, was taken from [38] and inflated to 2015 price.

Psychological intervention costs were calculated by combining therapists’ time (as described in the relevant source RCTs) with respective national unit costs [39]. All therapists were assumed to be Band 7 therapists according to the NHS Agenda for Change for qualified Allied Health Professionals. An initial GP visit for referral to psychological services was also considered. No booster sessions were assumed as there is no evidence to indicate that these are necessary for psychological treatment effect sustainment, and they are not routinely offered in clinical practice. Self-help intervention costs included the cost of either a book or a computerised programme and related infrastructure or equipment required for the programme delivery (license fee or website hosting, personal computers and capital overheads). The intervention cost of wait list was zero. Estimated psychological intervention costs for self-help interventions ranged between £205-£912 per person; for group therapies £551-£1,148 per person; and for individually delivered therapies £1,588-£2,359 per person (Table 2).

**Table 2:** Intervention costs of psychological treatments (2015 prices).

Intervention	Resource use details	Total cost per person^a^
SHNS, book	75 minutes contact with therapist plus cost of book (Rapee’s *Overcoming Shyness and Social Phobia*: *A Step by Step Guide* current cost on Amazon: £20.66)	£205
SHNS, internet	75 minutes contact with therapist; the annual cost of internet hosting of a self-help internet pilot programme in the UK is £14,000 (GDG information) divided by 30 people with social phobia expected to take up the programme in an IAPT service annually (IAPT audit of activity data provided by GDG); cost of hardware & capital overheads £13/person (2015 price, based on [40])	£664
SHWS, book	210 minutes contact with therapist plus cost of book as above	£453
SHWS, internet	210 minutes contact with therapist plus cost of internet hosting, hardware and capital overheads as above	£912
Exposure	12 group sessions x 2.5 hours each, 2 therapists & 6 participants per group = 10 therapist hours per service user	£1,148
Mindfulness training	8 group sessions x 2.5 hours each plus an all-day retreat (7.5 hours), 2 therapists & 12 participants per group = 4.58 therapist hours per service user	£551
GCBT, Heimberg	12 group sessions x 2.5 hours, 2 therapists & 6 participants per group = 10 therapist hours per service user	£1,148
GCBT, general	15 group sessions x 2 hours each, 2 therapists & 6 participants per group = 10 therapist hours per service user	£1,148
IPT	18 individual sessions x 50 min each = 15 therapist hours per service user	£1,698
PDPT	25 individual sessions x 50 min each = 20.83 therapist hours per service user	£2,341
Supportive therapy	14 individual sessions x 1 hour each = 14 therapist hours per service user	£1,588
ICBT, Hope	16 individual sessions x 1 hour each, with the exception of the first in-session exposure session which lasts 1.5 hours = 16.5 therapist hours per service user	£1,864
ICBT, C&W	14 individual sessions x 90 min each = 21 therapist hours per service user	£2,359
ICBT, short	14 individual sessions x 75 min each = 17.5 therapist hours per service user	£1,974
ICBT, general	16 individual sessions x 1 hour each = 16 therapist hours per service user	£1,809
Wait list	No related resource use	£0

^a^Cost of therapists was estimated using the unit cost of Band 7 qualified clinical psychologists (NHS Agenda for Change for qualified Allied Health Professionals), which includes salary, on-costs and overheads [39]; additional qualification costs estimated as a proportion of this unit cost, after examining unit costs without/with qualification costs for other mental health professionals (consultant psychiatrists and mental health nurses); estimated unit cost for Band 7 therapist equals £110 per hour; total cost per person includes a GP visit for referral to the psychological service; GP unit cost taken from [38].

C&W: Clark and Wells model; GCBT: group cognitive behavioural therapy; ICBT: individually delivered cognitive behavioural therapy; IPT: interpersonal therapy; PDPT: psychodynamic psychotherapy; SHNS: self-help no support; SHWS: self-help with support

Adults with social anxiety disorder incur costs to health and personal social services that are associated with their disorder. Annual health and personal social service costs associated with social anxiety disorder were taken from a published analysis [6] of service use data obtained from a British Psychiatric Morbidity Survey conducted in 1993–1994 [41]. These costs included GP consultations, home visits, counselling or therapy contacts and inpatient and outpatient secondary care, and were reported separately for people with social anxiety disorder and people without psychiatric morbidity (the latter was assumed to correspond to the state of ‘no social anxiety disorder’). These costs were not applied during the period of initial treatment (over which intervention costs were incurred), to avoid doublecounting of treatment costs, which might have already been included in the estimation of health and personal social service costs associated with the disorder, but were applied immediately after completion of initial treatment, at the end of 12 weeks.

Costs were expressed in 2015 prices, inflated, where necessary, using the Hospital and Community Health Services pay and prices index [38]. The inflation index for year 2015 was estimated using the average value of the Hospital and Community Health Services pay and prices indices of the previous 3 years. Costs and QALYs were discounted at 3.5% annually, following NICE guidance [42].

An overview of methods, data sources and a list of the key assumptions underpinning the economic model are provided in S2 File.

### Handling uncertainty

To account for the uncertainty around the input parameter point estimates, a probabilistic analysis was undertaken, in which input parameters were assigned probabilistic distributions [43]. Subsequently, 10,000 iterations were performed, each drawing random values out of the distributions fitted onto the model input parameters. Mean costs and QALYs and the Net Monetary Benefit (NMB) for each treatment option were calculated by averaging across the 10,000 iterations. The cost-effectiveness acceptability frontier was also plotted; this shows the treatment option with the highest mean NMB over different cost effectiveness thresholds, and the probability that the option with the highest NMB is the most cost-effective among those assessed. This method of presenting uncertainty has advantages over confidence interval estimation for incremental cost-effectiveness ratios, in particular in comparisons of multiple competing interventions, and presents uncertainty in a format that is more relevant to decision making [44].

The distributions of the probability of recovery for each intervention at end of treatment were defined directly from values recorded in the 10,000 iterations of the NMA. The log-odds of recovery on wait list was assumed to follow a normal distribution. The log-odds ratios of recovery for each treatment relative to wait list were applied to simulated values of this normal distribution and converted onto the probability scale. This ensured that the full posterior distribution of the relative treatment effects was used to estimate the absolute probabilities of recovery for each treatment [45].

Other probabilities and utility scores were assigned beta distributions. The risk ratio of relapse was assigned a log-normal distribution. Annual health and personal social service costs were assigned a gamma distribution. The estimation of distribution ranges was based on respective available data reported in the published sources of evidence.

Uncertainty in intervention costs was considered by assigning probability distributions around the number of GP visits and therapist sessions in individiually delivered psychological interventions, determined by completion rates and compliance data reported in RCTs included in the NMA. The same distributions around the number of GP visits were used for all pharmacological interventions. The number of therapist sessions per person attending group psychological interventions was not assigned a probabilistic distribution because the number of group sessions remains the same, whether a participant attends the full course of treatment or a lower number of sessions. Drug acquisition costs are not subject to uncertainty and thus were also not assigned probabilistic distributions. However, if people receiving pharmacological therapy attended considerably fewer GP visits than the mode, then they were assumed to be prescribed smaller amounts of medication than optimal, and to subsequently incur lower drug acquisition costs (either at initial or during maintenance treatment). Uncertainty in intervention costs of self-help interventions was considered by applying a normal distribution around the time spent with the therapist.

Table 3 reports the values of all model input parameters and provides details on the types and range of distributions assigned to each.

**Table 3:** Input parameters utilised in the economic model of interventions for adults with social anxiety disorder.

Input parameter	Mean value	Probabilistic distribution	Source of data—comments
**Probability of recovery, all interventions—end of treatment**	See Table 4	Distribution based on NMA	NMA; distribution formed by 10,000 iterations
**Annual probability of recovery, all interventions—years 2–5**	0.0377	Beta distribution on 12-year probability: α = 65; β = 111	[21]
**Annual probability of relapse, drugs—year 1**	0.4169	Midpoint between 2 beta distributions:	Midpoint between pooled relapse rate from drug arms and pooled relapse rate from pill placebo arms of 5 relapse prevention RCTs identified by a systematic literature search [26–30]
		α = 107; β = 293	
		α = 222; β = 170	
**Risk ratio of relapse, drugs versus psychological interventions—year 1**	3.00	Log-norm distribution	[31]
		95% CIs: 0.73 to 12.39	
**Annual probability of relapse, allinterventions—years 2–5**	0.0409	Beta distribution on 12-year	[21]
		probability: α = 26; β = 40	
**Utilities**		Beta distribution	
Recovery (no social anxiety disorder)	0.866	α = 4572; β = 707	Estimated using method of moments, based on published data in [33]
Non-recovery, relapse (social anxiety disorder)	0.659	α = 40; β = 20	
**Intervention resource use and costs**			
**Drug acquisition costs & health professional unit costs**	See Tables 1 & 2	No distribution assigned	
**Number of GP visits assigned to pharmacological interventions**		Different probabilities assigned to different numbers of sessions	Number of visits based on expert opinion; estimated probabilities based on completion rates reported in large pharmacological RCTs included in NMA (N>100) and further assumptions. If number of GP visits in initial treatment equalled 1 or 2, no maintenance treatment followed. If number of GP visits in initial treatment equalled 1, only 50% of the 12-week drug acquisition costs were incurred; if number of GP visits equalled zero in maintenance treatment, no 26-week drug acquisition costs were considered
Initial treatment (12 weeks)	4	65%: 4; 10%: 3, 5 or 6; 25%: 1 or 2	
Maintenance treatment (26 weeks)	3	55%: 3; 45%: 0 or 1 or 2 or 4	
**Number of sessions in individually delivered psychological interventions**		Different probabilities assigned to different numbers of session	
IPT	18	70%: 18; 15%: 14–17; 15%: 1–13	Number of sessions and estimated
PDPT	25	70%: 25; 15%: 21–24; 15%: 1–20	probabilities based on number of sessions
Supportive therapy	14	70%: 14; 15%: 10–13; 15%: 1–9	and completion rates reported in
ICBT, general	16	70%: 16; 15%: 12–15; 15%: 1–11	respective RCTs included in NMA and
ICBT, Hope	16	70%: 16; 15%: 12–15; 15%: 1–11	further assumptions.
ICBT, C&W	14	80%: 14; 20%: 10–13	
ICBT, short	14	70%: 14; 15%: 10–13; 15%: 1–9	
**Number of sessions in group psychological interventions**	As in Table 2	No distribution assigned	Participants missing one or more sessions assumed not to be replaced by others; therefore changes in number of sessions did not affect total intervention cost.
**Time spent with therapist in self-help (minutes)**		Normal distribution	Mean contact time based on data reported in relevant clinical studies; distributionbased on assumption
SHNS (book or internet)	75	SD: 0.3 of the mean	
SHWS (book or internet)	210	SD: 0.3 of the mean	
**Annual health and social care cost**		Gamma distribution	
Recovery (no social anxiety disorder)	£645	SE: £93	[6]
Non-recovery, relapse (social anxiety disorder)	£1,037	SE: £209	[6]
**Annual discount rate**	0.035	No distribution assigned	[42]

C&W: Clark and Wells model; ICBT: individually delivered cognitive behavioural therapy; IPT: interpersonal therapy; NMA: network meta-analysis; PDPT: psychodynamic psychotherapy; SHNS: self-help no support; SHWS: self-help with support

Deterministic sensitivity analyses explored the following alternative scenarios:

- self-help interventions to be supported by a Band 5 therapist and group therapies to be delivered by one Band 7 and one Band 6 therapists (in the base-case analysis all therapists were in Band 7 according to the NHS Agenda for Change for qualified Allied Health Professionals, with a unit cost of £110 per hour; Band 6 and Band 5 therapist unit costs were £93 and £87 per hour, respectively) [38,39].
- use of an alternative set of utility scores, based on EQ-5D data [40] derived from a community-based mental health European survey [46]. These data reflected a smaller difference in utility between social anxiety disorder and no mental disorder compared with those used in the base-case analysis (this scenario used utility values of 0.79 and 0.91 for social anxiety disorder and no social anxiety disorder, respectively).
- use of alternative time horizons of 1, 3, and 10 years following the 12 weeks of initial treatment. These scenarios were tested to explore the short-term cost effectiveness of interventions for social anxiety disorder and potential changes in their relative cost effectiveness over time and in the long term.
- the risk ratio of relapse of drugs versus psychological interventions for year 1 to equal a range of values between 1.0 and 2.0, as this may be closer to real life than the reported mean value of 3.0 [31] that was utilised in the economic analysis.

## Results

### Output of network meta-analysis used in the economic analysis

Results of the NMA that populated the economic model are provided in Table 4, which shows the probability of recovery at end of treatment for each intervention. Treatments have been ranked from the most to the least effective; the mean probability of recovery ranged from 0.62 (95% credible intervals 0.16 to 0.95) for ICBT C&W down to 0.10 (95% credible intervals 0.01 to 0.39) for wait list. Overall, the various forms of ICBT showed high probabilities of recovery (mean value in the range of 0.39–0.62) with other individually delivered psychological therapies being less effective (range of mean probabilities of recovery 0.16–0.26). The mean probabilities of recovery for drugs and self-help interventions were in the range of 0.27 to 0.40, with the exception of phenelzine, the mean probability of which reached 0.51 (second most effective option following ICBT C&W). The mean probabilities of recovery for group psychological therapies ranged from 0.19 (mindfulness) to 0.34 (GCBT general).

**Table 4:** Results of network meta-analysis that were utilised in the economic model: probability of recovery at end of treatment. Interventions ranked according to probability of recovery (highest to lowest).

Intervention	Probability of recovery		
	mean	median	95% credible intervals
ICBT, C&W	0.62	0.65	0.16 to 0.95
Phenelzine	0.51	0.51	0.09 to 0.91
ICBT, general	0.47	0.46	0.08 to 0.90
ICBT, Hope	0.41	0.38	0.06 to 0.86
Paroxetine	0.40	0.37	0.06 to 0.85
ICBT, short	0.39	0.36	0.05 to 0.84
Venlafaxine	0.38	0.35	0.05 to 0.84
Fluvoxamine	0.37	0.34	0.05 to 0.84
Sertraline	0.37	0.33	0.04 to 0.83
Escitalopram	0.35	0.31	0.04 to 0.82
SHWS, internet	0.35	0.31	0.05 to 0.80
Fluoxetine	0.35	0.31	0.04 to 0.81
SHWS, book	0.34	0.30	0.04 to 0.81
GCBT, general	0.34	0.30	0.04 to 0.80
SHNS, book	0.34	0.30	0.04 to 0.80
Citalopram	0.34	0.29	0.04 to 0.82
Exposure	0.33	0.29	0.04 to 0.79
Mirtazapine	0.33	0.28	0.03 to 0.83
GCBT, Heimberg	0.32	0.28	0.04 to 0.78
Moclobemide	0.30	0.25	0.03 to 0.76
Pregabaline	0.29	0.25	0.03 to 0.77
SHWS, book	0.27	0.22	0.03 to 0.73
PDPT	0.26	0.21	0.03 to 0.72
Pill placebo	0.21	0.16	0.02 to 0.64
IPT	0.20	0.15	0.02 to 0.64
Mindfulness	0.19	0.14	0.02 to 0.63
Supportive therapy	0.16	0.11	0.01 to 0.57
Wait list	0.10	0.07	0.01 to 0.39

The log-odds of recovery on wait list was assumed to follow a normal distribution with mean -2.629 and variance 1.235 (estimated using all the wait list arms of RCTs included in the NMA); this translates into a probability of recovery for wait list (mean, 95% credible intervals) as shown above.

C&W: Clark and Wells model; GCBT: group cognitive behavioural therapy; ICBT: individually delivered cognitive behavioural therapy; IPT: interpersonal therapy; NMA: network meta-analysis; PDPT: psychodynamic psychotherapy; SHNS: self-help no support; SHWS: self-help with support

### Cost effectiveness

ICBT C&W was the most cost-effective intervention for adults with social anxiety disorder, with the highest NMB at the NICE lower cost effectiveness threshold of £20,000/QALY [47], despite having the highest intervention cost. The second most cost-effective option was ICBT general, while other forms of ICBT, i.e. ICBT Hope and ICBT short ranked 6^th^ and 9^th^, respectively. Phenelzine ranked 3^rd^, while book-based self-help without / with support ranked 4^th^ and 5^th^, respectively. Group-based psychological interventions were not particularly cost-effective relative to other treatments, ranking in places between 11^th^ and 17^th^, except mindfulness training, which ranked 23^rd^. Drugs (apart from phenelzine) also tended to rank low in terms of cost effectiveness, taking up places between 8^th^ and 22^nd^; following phenelzine, the order of the next most cost-effective drugs was: paroxetine, venlafaxine, fluvoxamine, sertraline, escitalopram and fluoxetine. Internet-based self-help ranked 7^th^ (with support) and 20^th^ (without support). Psychodynamic psychotherapy ranked 25^th^, just above wait list; interpersonal psychotherapy ranked 27^th^ while supportive therapy was the least cost-effective intervention, ranking in the last (28^th^) place. Table 5 provides the mean total costs and QALYs per person and the percentage of adults that are well (no social anxiety disorder) at 5 years for each intervention assessed in the economic analysis; it also provides the results of incremental analysis, the mean NMB of each intervention at the NICE lower cost effectiveness threshold (£20,000/QALY), and the ranking of interventions according to their mean NMB. Detailed probabilistic results, including 95% credible intervals of costs, QALYs and NMB of each intervention, the mean ranking of each intervention and its probability of being cost-effective are provided in S1 Table.

**Table 5:** Cost effectiveness of interventions for adults with social anxiety disorder: results of probabilistic analysis. Mean values per person 5 years after end of treatment.

Intervention	% without SA at 5 years	Mean QALYs	Mean total costs (£)	Incremental analysis & ICERs (£/QALY)	Mean NMB (£)	Ranking by highest NMB
ICBT, C&W	50.25	3.75	6,178	9,179 versus phenelzine	68,810	1
ICBT, general	41.40	3.64	5,714	Extendendly dominated^a^	67,040	2
ICBT, Hope	37.52	3.59	5,853	Dominated	65,916	6
ICBT, short	36.25	3.57	5,970	Dominated	65,479	9
Phenelzine	34.80	3.57	4,557	1,472 versus SHNS book	66,899	3
SHWS, internet	34.02	3.54	5,186	Dominated	65,699	7
SHWS, book	33.59	3.54	4,741	Dominated	66,037	5
GCBT, general	33.54	3.54	5,436	Dominated	65,327	11
SHNS, book	33.28	3.53	4,501		66,197	4
Exposure	33.00	3.53	5,448	Dominated	65,179	14
GCBT, Heimberg	32.40	3.52	5,463	Dominated	65,013	17
Paroxetine	30.01	3.51	4,561	Dominated	65,603	8
Venlafaxine	29.46	3.50	4,633	Dominated	65,380	10
Fluvoxamine	29.08	3.50	4,666	Dominated	65,245	12
Sertraline	28.75	3.49	4,583	Dominated	65,239	13
SHNS, internet	29.55	3.49	5,042	Dominated	64,714	20
Escitalopram	28.12	3.48	4,591	Dominated	65,058	15
Fluoxetine	27.98	3.48	4,597	Dominated	65,016	16
PDPT	28.80	3.48	6,509	Dominated	63,055	25
Citalopram	27.53	3.47	4,602	Dominated	64,890	18
Mirtazapine	27.18	3.47	4,611	Dominated	64,786	19
Moclobemide	25.95	3.45	4,740	Dominated	64,323	21
Pregabalin	25.78	3.45	4,898	Dominated	64,118	22
IPT	25.44	3.44	5,987	Dominated	62,728	27
Mindfulness	24.86	3.43	5,041	Dominated	63,527	23
Pill placebo	22.29	3.40	4,713	Dominated	63,360	24
Supportive therapy	22.89	3.40	5,934	Dominated	62,136	28
Wait list	20.43	3.37	4,593	Dominated	62,810	26

^a^Extended dominance occurs when an option is less effective and more costly than a linear combination of two alternative options.

Interventions have been ranked from the most to least effective according to the number of QALYs gained.

C&W: Clark and Wells model; GCBT: group cognitive behavioural therapy; ICBT: individually delivered cognitive behavioural therapy; ICER: Incremental Cost Effectiveness Ratio; IPT: interpersonal therapy; NMB: Net Monetary Benefit, estimated using a willingeness to pay £20,000/QALY; PDPT: psychodynamic psychotherapy; SA: Social Anxiety disorder; SHNS: self-help no support; SHWS: self-help with support.

**Fig 2:**
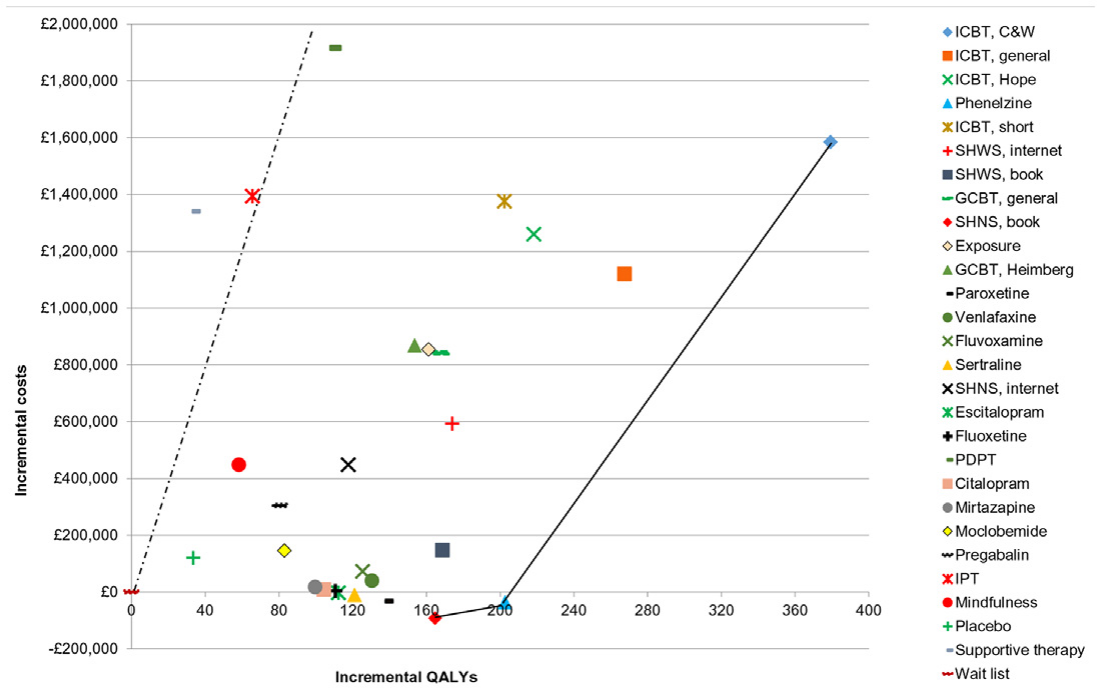
Cost-effectiveness plane showing the incremental costs and QALYs of all interventions versus wait list. Wait list is placed at the origin; results are for 1,000 adults with social anxiety disorder at 5 years after treatment. The continuous line shows the cost-effectiveness efficiency frontier, while the slope of the dotted line indicates the NICE lower cost effectiveness threshold (£20,000/QALY). The data used to construct Fig 2 are provided in Table 5.

The cost effectiveness plane (Fig 2) shows the incremental costs and QALYs of all interventions versus wait list (placed at the origin). The cost-effectiveness efficiency frontier (continuous line) links all interventions that are not dominated by absolute or extended dominance and are thus cost-effective at different cost effectiveness thresholds. The slope of the dotted line indicates the NICE lower cost effectiveness threshold, suggesting that IPT and supportive therapy are not cost-effective compared with wait list (since both lie on the left of the dotted line).

**Fig 3:**
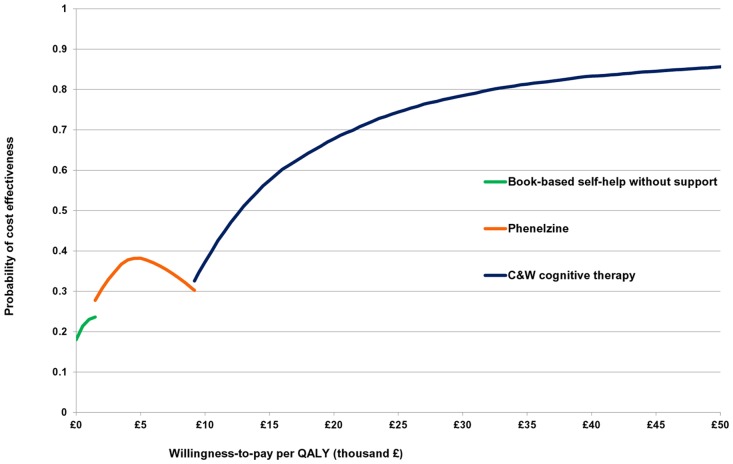
Cost-effectiveness acceptability frontier of pharmacological and psychological interventions for adults with social anxiety disorder. The values used to construct Fig 3 are provided in S2 Table.

According to the cost-effectiveness acceptability frontier (Fig 3), for cost effectiveness thresholds between zero and £1,472/QALY, book-based self-help without support has the highest NMB (i.e. it is the most cost-effective treatment), but its probability of being cost-effective does not exceed 0.24. Phenelzine is the most cost-effective intervention for cost effectiveness thresholds between £1,472/QALY and £9,179/QALY, with a maximum probability of cost effectiveness just above 0.38. At even higher cost effectiveness thresholds, ICBT (C&W) has the highest NMB and a probability of cost effectiveness that increases with increasing cost effectiveness thresholds. The probability of ICBT (C&W) being cost-effective reaches 0.68 at the NICE £20,000/QALY threshold of and goes beyond 80% when the threshold exceeds £32,500/QALY.

Results were robust to use of lower unit costs for therapists involved in self-help and group psychological interventions and the use of more conservative utility data. ICBT (C&W) remained the most cost-effective intervention under these scenarios, and the cost effectiveness ranking of interventions was broadly the same. Sensitivity analysis of different time horizons indicated that, in the short-term, pharmacological interventions are overall more cost-effective than psychological interventions. At a 1-year time horizon, phenelzine produced the highest NMB and was the most cost-effective among treatment options, whereas ICBT (C&W) ranked only 17^th^. Of the first 10 places in ranking, 8 were occupied by pharmacological interventions and the remaining 2 were occupied by book-based self-help (without / with support). However, by 3 years ICBT (C&W) became the most cost-effective intervention followed by phenelzine. The relative cost effectiveness of drugs was reduced compared with their cost effectivess at a 1-year time horizon; in constrast, the cost effectiveness of psychological interventions improved, whereas the cost effectiveness of book-based self-help remained broadly the same. By 10 years, the ranking of interventions by cost effectiveness mirrored their ranking by effectiveness (number of QALYs), with the 4 individual forms of CBT occupying the 4 first places in ranking due to their higher intervention costs having been fully offset by health and personal social service cost-savings.

As expected, use of lower risk ratios of relapse for drugs versus psychological interventions favoured drugs. When an equal risk of relapse was assumed, phenelzine became the most cost-effective intervention and, with the exception of ICBT (C&W) which ranked 2^nd^, the rest top-5 places in cost effectiveness ranking were occupied by drugs. However, ICBT (C&W) became the most cost-effective option when the risk ratio of relapse rose at just 1.15, and performance of psychological interventions improved substantially with increasing values of this ratio. For example, at a value of 1.5, 3 psychological interventions (ICBT, C&W; ICBT, general and SHNS, book) ranked in the top-5 places, while at a risk ratio of 2, the 5 most cost-effective options included 4 psychological interventions and phenelzine, with overall results being broadly consistent with those of the base-case analysis.

Results of deterministic one-way sensitivity analyses are provided in S3 File. The deterministic ranking of interventions according to cost effectiveness for different time horizons tested is provided in S3 Table. The deterministic ranking of interventions according to cost effectiveness at different values of the risk ratio of relapse of drugs versus psychological interventions (year 1) is shown in S4 Table.

## Discussion

Individual forms of CBT appear to be the most cost-effective interventions for adults with social anxiety disorder. Self-help, in particular book-based, is cost-effective compared with other options. Group-based psychological interventions and drugs (with the exception of phenelzine, which was among the most cost-effective options) do not appear to be particularly cost-effective relative to other treatments. Other individually delivered psychological interventions such as psychodynamic psychotherapy, interpersonal therapy and supportive therapy are the least cost-effective options for adults with social anxiety disorder. The emergence of individual forms of CBT as most cost-effective options despite their high intervention costs is attributed to two factors: their higher effectiveness compared with other interventions and the considerably lower risk of relapse of psychological interventions compared with drugs; consequently these interventions result in higher health benefits (QALYs), but also in a reduction in health and personal social service costs attributable to social anxiety disorder. Moreover, the cost effectiveness of individual forms of CBT appears to increase over time because their high intervention costs (which are responsible for the low performance of these interventions 1 year after treatment) are spread over a longer time period and are offset to a greater extent by health and personal social service cost savings associated with the successful management of social anxiety disorder.

### Strengths and limitations

The bigest strength of our analysis is the utilisation of efficacy data derived from a systematic literature review and NMA. This methodology enabled us to consider information from both direct and indirect comparisons between interventions, and allowed simultaneous inference on all treatment options examined in trial pair-wise comparisons while preserving randomisation [20,48]. This approach for evidence synthesis is essential for populating model-based economic studies assessing more than two competing interventions. The NMA principally utilised continuous data to estimate the relative treatment effects of interventions, and then transformed the estimated SMDs into probabilities of recovery. Such a transformation is valid as long as the assumed relationship between the treatment effect based on continuous data and the treatment effect estimated using recovery data holds. This assumption could not be checked for all interventions, but available data indicated a strong relationship and therefore this transformation is unlikely to have introduced substantial bias into the analysis [14]. These assumptions along with the limitations of the NMA model and the limitations of the RCTs considered in the NMA [14] may have impacted on the quality of the respective input parameters used to populate the economic model.

A number of interventions such as benzodiazepines, anticonvulsants and combination therapies that were included in the NMA [14] were not considered in the economic analysis. Benzodiazepines cannot be used beyond 2–4 weeks for the treatment of anxiety [36]. Clinical evidence on anticonvulsants and combination therapies was particularly limited and overall of low quality [14] so that inclusion of these interventions in the analysis would not have important implications for decision making.

An important limitation of the economic analysis was the poor quality of the relapse data used, due to lack of robust evidence on the relative risk of relapse between pharmacological and psychological interventions for social anxiety disorder. Furthermore, due to lack of intervention-specific data, the economic model assumed one (common) risk of relapse applied to all pharmacological interventions, and one (common) risk of relapse across all psychological ones. Nevertheless, evidence suggests that, in constrast to pharmacological interventions, which are characterised by a relatively high relapse risk at 6 months of maintenance treatment [26–30], the effect of psychological interventions is well-maintained in the long-term, after end of treatment [49,50]. Moreover, the mean probabilities of relapse for drugs and psychological interventions estimated for the economic model (42% versus 14%, respectively) are similar to relapse rates reported in trials of these types of therapies for social anxiety disorder, very close to respective relapse rates reported for people with obsessive compulsive disorder (45% versus 12%, respectively) [51] and broadly consistent with respective figures reported for panic disorder (40% versus 5%, respectively) [52]. Sensitivity analysis showed that more conservative assumptions regarding the superiority of psychological interventions over drugs in retaining long-term effects would lead to broadly the same conclusions.

Another limitation of the economic analysis was the lack of consideration of the impact of the side effects of drugs on the HRQoL and costs, due to inconsistent reporting of side effect data in the RCTs included in the NMA. In particular, use of phenelzine, which was ranked 3^rd^ most cost-effective intervention, is associated with a potentially dangerous interaction with foods containing tyramine that may lead to episodes of high blood pressure, thus imposing dietary restrictions to people administered this drug. Omission of the impact of drug-related side effects from the economic model has likely overestimated the cost effectiveness of all pharmacological interventions. Other limitations of the model include the use of utility data that were not specific to health states of social anxiety disorder and the use of resource use data associated with social anxiety disorder collected almost 20 years ago, due to lack of better quality and more up-to-date data, respectively.

### Comparison with other published studies in the field

Published economic analyses have explored the cost-effectiveness of a very limited range of interventions for social anxiety disorder and concluded that escitalopram [53], group CBT [54–56] and computer-based self-help [55–57] are cost-effective options. All studies were characterised by methodological limitations, such as exclusive consideration of intervention costs and omission of other relevant healthcare [54,57] or equipment (computer) [55–57] costs, an inability to accurately record relevant healthcare costs from all study participants [53], and inappropriate estimation of the relative effectiveness of interventions, by utilising their effect sizes versus different controls (waitlist or pill placebo), so that the effect sizes were not comparable [54]. Therefore, these findings should be treated with caution.

### Generalisability of the results and implications of the study

Our analysis has been conducted from the perspective of the British NHS and Personal Social Services. Results are likely generalisable to other settings with similar funding and structure of healthcare and personal social services and comparable care pathways for social anxiety disorder. It should be noted that conclusions on cost effectiveness ultimately rely on the cost effectiveness threshold adopted, and this depends on the policy makers’ willingness-to-pay for treatment benefits, which may vary across countries and health systems. The economic model did not incorporate productivity losses. However, more effective interventions can reasonably be expected to lead to improved functioning, and, in turn, to increased employment rates and reduced days of sick leave, thus to an increase in productivity gains. Consequently, consideration of productivity losses in our analysis would probably further favour individual forms of CBT.

Based on our findings and after considering potential drug interactions (fluvoxamine), side effects that were not considered in the model (such as possible blood pressure changes with venlafaxine and phenelzine, discontinuation symptoms associated with venlafaxine and SSRIs and in particular with paroxetine), dietary restrictions with the MAOIs, the limited evidence base for fluoxetine relative to other drugs, and concerns about the quality of phenelzine data which may have led to overestimation of the drug’s effect, the NICE clinical guideline on social anxiety disorder recommended individually delivered CBT that has been specifically designed for social anxiety disorder as first line treatment for adults with social anxiety disorder [15]. Supported self-help was recommended as second-line psychological treatment, while SSRIs (escitalopram or sertraline) were recommended for people preferring pharmacological treatment. Additional recommendations were made for people not responding or partially responding to treatment and for those developing side effects. Further good quality research is needed on the comparative long-term outcomes of psychological and pharmacological interventions as well as on costs and utility values associated with social anxiety disorder, in order to establish the relative cost effectiveness of interventions for social anxiety disorder with greater certainty.

## Supporting Information

S1 File Outcome measures used in the clinical analysis. (DOCX)

S2 File Overview of methods, data sources and key assumptions underpinning the economic model. (DOCX)

S3 File Results of deterministic one-way sensitivity analyses. Mean values per person 5 years after end of treatment
(DOCX)

S1 Table Detailed results of probabilistic sensitivity analysis. Mean values (95% credible intervals) per person, 5 years after end of treatment
(DOCX)

S2 Table Values used to construct Fig 3. Probability of cost effectiveness of the interventions with the highest net monetary benefit at varying levels of willingness-to-pay per QALY gained.
(DOCX)

S3 Table Ranking of interventions by cost effectiveness at different time horizons of the economic model—deterministic analysis. (DOCX)

S4 Table Ranking of interventions by cost effectiveness at different values of the risk ratio of relapse of drugs versus psychological interventions (year 1)–deterministic analysis. (DOCX)

## Abbreviations

CBT: Cognitive behavioural therapy
GDG: Guideline Development Group
HRQoL: Health-Related Quality of Life
NICE: National Institute for Health and Care Excellence
NMA: Network meta-analysis
NMB: Net Monetary Benefit
