# Supplementary material for: The Cost Effectiveness of Psychological and Pharmacological Interventions for Social Anxiety Disorder: A Model-Based Economic Analysis
Source: PLoS One. 2015 Oct 27;10(10):e0140704. doi: 10.1371/journal.pone.0140704 (PMC4624770; doi:10.1371/journal.pone.0140704)
Supplement: S1 File — (DOCX) [file pone.0140704.s001.docx]

**Outcome measures used in the clinical analysis**

Continuous

Anxiety Disorders Interview Schedule (ADIS-IV): Fear and Avoidance [1]

Brief Social Phobia Scale [2,3]

Clinical Global Impression (CGI): Severity [4]

Fear of Negative Evaluation Scale [5]

Fear Questionnaire (FQ): Social Phobia [6]

Liebowitz Social Anxiety Scale [7]

Liebowitz Social Anxiety Scale –Self Report [8,9]

Social Avoidance and Distress Scale (SADS) [5]

Social Interaction Anxiety Scale (SIAS); Social Phobia Scale (SPS) [10]

Social Phobia Anxiety Inventory [11]

Dichotomous

Recovery (no longer meeting criteria for diagnosis of social anxiety disorder)

*References*

1. Brown TA, Di Nardo P, Barlow DH. Anxiety disorders interview schedule for DSM-IV: client interview schedule. Oxford: Oxford University Press; 1994.
2. Davidson JR, Miner CM, De Veaugh-Geiss J, Tupler LA, Colket JT, Potts NL. The Brief Social Phobia Scale: a psychometric evaluation. Psychol Med 1997;27: 161-166.
3. Davidson JR, Potts NL, Richichi EA, Ford SM, Krishnan KR, Smith RD, et al. The Brief Social Phobia Scale. J Clin Psychiatry 1991;52 (suppl): 48-51.
4. Guy W. The Clinical Global Impression Scale. ECDEU assessment manual for psychopharmacology-revised. Rockville: US Department of Health, Education and Welfare, ADAMHA, MIMH Psychopharmacology Research Branch; 1976. pp 218-222.
5. Watson D, Friend R. Measurement of social-evaluative anxiety. J Consult Clin Psychol 1969;33: 448-457.
6. Marks IM, Mathews AM. Brief standard self-rating for phobic patients. Behav Res Ther 1979;17: 263-267.
7. Heimberg RG, Horner KJ, Juster HR, Safren SA, Brown EJ, Schneier FR, et al. Psychometric properties of the Liebowitz Social Anxiety Scale. Psychol Med 1999;29: 199-212.
8. Fresco DM, Coles ME, Heimberg RG, Liebowitz MR, Hami S, Stein MB, et al. The Liebowitz Social Anxiety Scale: a comparison of the psychometric properties of self-report and clinician-administered formats. Psychol Med 2001;31: 1025-1035.
9. Baker SL, Heinrichs N, Kim HJ, Hofmann SG. The Liebowitz social anxiety scale as a self-report instrument: a preliminary psychometric analysis. Behav Res Ther 2002;40: 701-715.
10. Mattick RP, Clarke JC. Development and validation of measures of social phobia scrutiny fear and social interaction anxiety. Behav Res Ther 1998;36: 455-470.
11. Beidel DC, Turner SM, Stanley MA, Dancu CV. The Social Phobia and Anxiety Inventory: concurrent and external validity. Behav Ther 1989;20: 417-427.
