# Supplementary material for: The Cost Effectiveness of Psychological and Pharmacological Interventions for Social Anxiety Disorder: A Model-Based Economic Analysis
Source: PLoS One. 2015 Oct 27;10(10):e0140704. doi: 10.1371/journal.pone.0140704 (PMC4624770; doi:10.1371/journal.pone.0140704)
Supplement: S2 File — (DOCX) [file pone.0140704.s002.docx]

**Overview of methods, data sources and key assumptions** **underpinning the economic model**

**Details and assumptions in the model structure and in the estimation of clinical input parameters**

1. **Decision-tree component**
2. The study population entering the decision-tree comprised adults aged at least 18 years who fulfilled diagnostic criteria for social anxiety disorder and were eligible for all the treatment options included in the analysis.
3. The decision-tree consisted of initial treatment with one of the 28 interventions assessed followed by 1-year follow up.
4. For purposes of estimation of QALYs, initial treatment was assumed to last 12 weeks for all interventions; this modelling assumption did not affect estimated resource use associated with psychological interventions.
5. The total time spent by the study population in the decision tree was 12 weeks (initial treatment) plus 1 year (follow up).
6. After initial treatment, people either recovered, no further meeting criteria for diagnosis (being in a state of ‘no social anxiety disorder’) or failed to recover (remaining in the state of ‘social anxiety disorder’).
7. People recovering after pharmacological treatment were assumed to receive another 26 weeks of maintenace treatment with the same drug; people recovering after psychological treatment did not receive further maintenance treatment. Individuals not recovering were assumed to stop treatment.
8. In the 1-year follow-up, people who did not recover were assumed to remain in the ‘social anxiety disorder’ state. Those who recovered might relapse, meeting again diagnostic criteria for the disorder, and thus re-enter the ‘social anxiety disorder’ state. Relapse was assumed to occur at 6 months after completion of initial treatment (mid-point of the 1-year follow-up).
9. Efficacy of initial treatment for each intervention was derived from a systematic review and network meta-analysis (NMA) of interventions for adults with social anxiety disorder [1]. The NMA utilised continuous data (changes on symptom scales of social anxiety) expressed in the form of a standardised mean difference (SMD) and recovery data (loss of diagnosis at end of treatment). SMD data were adjusted using the identified relationship between SMD and recovery. Subsequently, SMDs of all interventions versus waitlist were transformed into log-odds ratios of recovery, and these in turn were used to estimate absolute probabilities of recovery for each intervention using the probability of recovery for wait list as baseline, which was calculated by pooling recovery data from all wait list arms in the RCTs included in the NMA.
10. The first-year probability of relapse after pharmacological maintenance treatment was assumed to equal the midpoint between the risk of relapse during pharmacological maintenance treatment and the risk of relapse of responders to drug treatment who received no maintenance treatment; relevant estimates were taken from pooling placebo-controlled pharmacological RCTs on relapse prevention in adults with social anxiety disorder [2–6]. This probability was applied to all pharmacological treatment arms of the model and to pill placebo.
11. The first-year probability of relapse after recovery with psychological interventions was calculated by dividing the first-year probability of relapse for drugs by the risk ratio of relapse of drugs versus psychological therapies; the latter was estimated from data reported in an observational 12-month follow-up study [7] of adults with social anxiety disorder that had responded to either phenelzine or group CBT [8]. This probability was applied to all psychologial treatment arms of the model.
12. The first-year probability of relapse after recovery in wait list was estimated using data from a 12-year naturalistic study on adults with mental health problems, including social anxiety disorder [9].
13. **Markov model component**
14. The Markov model included two states: ‘social anxiety disorder’ and ‘no social anxiety disorder’.
15. The Markov model component was run for 4 years, making the total time horizon of the analysis 12 weeks plus 5 years.
16. The Markov model was run in yearly cycles; a half-cycle correction was applied.
17. The transition probabilities between the two health states of the Markov model were based on recovery and relapse data on adults with social anxiety disorder reported in a 12-year naturalistic study [9]. These transition probabilities were applied to all model cohorts, regardless of initial treatment.

**Details and assumptions in the estimation of utility values used in the model**

1. The model was populated with utility data obtained from a Finish national health survey [10]. The study reported EQ-5D utility scores (estimated using the UK Time Trade-Off Tarrif) for people with social anxiety disorder and people with no mental disorder over the last 12 months (assessment was made using the Munich version of the Composite International Diagnostic Interview, M-CIDI).
2. Utility scores for people with social anxiety disorder over the previous 12 months were used for the ‘social anxiety disorder’ state. Utility scores for people with no mental disorder over the last 12 months were used as a proxy for the state of ‘no social anxiety disorder’.

**Details and assumptions in the estimation of costs used in the model**

1. The analysis adopted the perspective of the British National Health Service (NHS) and Personal Social Services. The price year was 2015. Costs (and benefits) were discounted at an annual rate of 3.5%.
2. Pharmacological intervention costs consisted of drug acquisition and general practitioner (GP) visit costs. The intervention cost of pill placebo comprised GP visit costs only. Initial drug treatment lasted 12 weeks, followed, in people who recovered, by 26 weeks of maintenance treatment of the same drug and daily dosage. Initial drug treatment included 4 GP visits; maintenance treatment for those recovering included 3 extra GP visits. Unit costs were taken from national sources [11,12].
3. Psychological intervention costs reflected therapists’ time. Therapists were assumed to be Band 7 qualified clinical psychologists (NHS Agenda for Change for qualified Allied Health Professionals). An initial GP visit for referral to psychological services was also considered. No maintenance treatment (booster sessions) was considered. Self-help intervention costs included the cost of either a book or a computerised programme and related infrastructure or equipment required for the programme delivery (license fee or website hosting, personal computers and capital overheads). The intervention cost of wait list was zero. Unit costs were taken from national sources [12,13].
4. All people in the model were assumed to incur costs to health and personal social services further to intervention costs. Health and personal social care costs for people with social anxiety disorder and people without psychiatric morbidity were taken from a published study of service use data [14] obtained from a British Psychiatric Morbidity Survey conducted in 1993–1994 [15]. Costs reported for people without psychiatric morbidity were applied to the state of ‘no social anxiety disorder’. These costs were not applied during the period of initial treatment, but were applied immediately after completion of initial treatment, at the end of 12 weeks.

**Details and assumptions in the estimation of the probabilistic distributions of the model input parameters**

1. The probabilistic model was run 10,000 times. Mean costs and QALYs and the Net Monetary Benefit (NMB) for each treatment option were calculated by averaging across the 10,000 iterations.
2. The distributions of the probability of recovery for each intervention at end of treatment were defined directly from values recorded in the 10,000 iterations of the NMA. The log-odds of recovery on wait list was assumed to follow a normal distribution. The log-odds ratios of recovery for each treatment relative to wait list were applied to simulated values of this normal distribution and converted onto the probability scale.
3. All probabilities and utility values were assigned a beta distribution.
4. The risk ratio of relapse (drugs versus psychological interventions) was assigned a log-normal distribution.
5. Annual health and personal social service costs were assigned a gamma distribution.
6. Probability distributions were applied around the number of GP visits; different probabilities were assigned to different numbers of GP visits so that the sum of probabilities equalled 1. The same distributions were used for all pharmacological interventions.
7. If people receiving pharmacological therapy attended considerably fewer GP visits than the mode, they were assumed to be prescribed smaller amounts of medication than optimal. If the number of GP visits in initial treatment equalled 1 or 2, no maintenance treatment followed. If the number of GP visits in initial treatment equalled 1, only 50% of the 12-week drug acquisition costs were incurred; if the number of GP visits equalled zero in maintenance treatment, no 26-week drug acquisition costs were considered.
8. Probability distributions were applied around the number of individually-delivered therapist sessions; different probabilities were assigned to different numbers of therapist sessions so that the sum of probabilities equalled 1. The distributions assigned were specific to each individually delivered psychological therapy.
9. The number of therapist sessions per person attending group psychological interventions was not assigned a probabilistic distribution.
10. Uncertainty in intervention costs of self-help interventions was considered by applying a normal distribution around the time spent with the therapist.
11. The estimation of distribution ranges was based on available data in the published sources of evidence and further assumptions, where relevant data were not available.

**References**

1. Mayo-Wilson E, Dias S, Mavranezouli I, Kew K, Clark DM, Ades AE, et al. Psychological and pharmacological interventions for social anxiety disorder in adults: a systematic review and network meta-analysis. Lancet Psychiatry 2014;1: 368-376.
2. Kumar R, Pitts C, Carpenter D. Response to paroxetine is maintained during continued treatment in patients with social anxiety disorder. Eur Neuropsychopharmacol 1999;9: S312-313.
3. Stein DJ, Versiani M, Hair T, Kumar R. Efficacy of paroxetine for relapse prevention in social anxiety disorder: a 24-week study. Arch Gen Psychiatry 2002;59: 1111-1118.
4. Montgomery SA, Nil R, Durr-Pal N, Loft H, Boulenger JP. A 24-week randomized, double-blind, placebo-controlled study of escitalopram for the prevention of generalized social anxiety disorder. J Clin Psychiatry 2005;66: 1270-1278.
5. Van Ameringen MA, Lane RM, Walker JR, Bowen RC, Chokka PR, Goldner EM, et al. Sertraline treatment of generalized social phobia: a 20-week, double-blind, placebo-controlled study. Am J Psychiatry 2001;158: 275-281.
6. Greist GH, Liu-Dumaw M, Schweizer E, Feltner D. Efficacy of pregabalin in preventing relapse in patients with generalized social anxiety disorder: results of a double-blind, placebo-controlled 26-week study. Int Clin Psychopharmacol 2011;26: 243-251.
7. Liebowitz MR, Heimberg RG, Schneier FR, Hope DA, Davies S, Holt CS, et al. Cognitive-behavioral group therapy versus phenelzine in social phobia: long-term outcome. Depress Anxiety 1999;10: 89-98.
8. Heimberg RG, Liebowitz MR, Hope DA, Schneier FR, Holt CS, Welkowitz LA, et al. Cognitive behavioral group therapy vs phenelzine therapy for social phobia: 12-week outcome. Arch Gen Psychiatry 1998;55: 1133-1141.
9. Bruce SE, Yonkers KA, Otto MW, Eisen JL, Weisberg RB, Pagano M, et al. Influence of psychiatric comorbidity on recovery and recurrence in generalized anxiety disorder, social phobia, and panic disorder: a 12-year prospective study. Am J Psychiatry 2005;162: 1179-1187.
10. Saarni SI, Suvisaari J, Sintonen H, Pirkola S, Koskinen S, Aromaa A, et al. Impact of psychiatric disorders on health-related quality of life: general population survey. Br J Psychiatry 2007;190: 326-332.
11. NHS Business Services Authority, NHS Prescription Services. Electronic Drug Tariff for England and Wales, May 2015. Compiled on behalf of the Department of Health; 2015. Available: http://www.nhsbsa.nhs.uk/PrescriptionServices/4940.aspx
12. Curtis L. Unit costs of health & social care 2014. Canterbury: Personal and Social Services Research Unit, University of Kent; 2014.
13. Curtis L. Unit costs of health & social care 2010. Canterbury: Personal and Social Services Research Unit, University of Kent; 2010.
14. Patel A, Knapp M, Henderson J, Baldwin D. The economic consequences of social phobia. J Affect Disord 2002;68: 221-233.
15. Meltzer H, Gill B, Petticrew M, Hinds K. OPCS surveys of psychiatric morbidity in great britain, report 2: physical complaints, service use and treatment of adults with psychiatric disorders. London: Office for National Statistics; 1995.
