# Supplementary material for: The Cost Effectiveness of Psychological and Pharmacological Interventions for Social Anxiety Disorder: A Model-Based Economic Analysis
Source: PLoS One. 2015 Oct 27;10(10):e0140704. doi: 10.1371/journal.pone.0140704 (PMC4624770; doi:10.1371/journal.pone.0140704)
Supplement: S3 File — Mean values per person 5 years after end of treatment (DOCX) [file pone.0140704.s003.docx]

**Results of deterministic sensitivity analyses.** Mean values per person 5 years after end of treatment

1. **base-case analysis**

| **Intervention** | **% without SA at 5 years** | **Mean QALYs** | **Mean total costs (£)** | **Incremental analysis & ICERs (£/QALY)** |  | **Mean NMB (£)** | **Ranking by highest NMB** |
| --- | --- | --- | --- | --- | --- | --- | --- |
| ICBT, C&W | 52.05 | 3.77 | 6,217 | 8,084 versus phenelzine |  | 69,144 | 1 |
| ICBT, general | 42.74 | 3.65 | 5,880 | Extendedly dominated^a^ |  | 67,150 | 2 |
| ICBT, Hope | 38.67 | 3.60 | 6,029 | Dominated |  | 65,982 | 6 |
| ICBT, short | 37.34 | 3.58 | 6,169 | Dominated |  | 65,509 | 10 |
| Phenelzine | 34.79 | 3.57 | 4,639 | 5,898 versus SHNS book |  | 66,819 | 3 |
| SHWS, internet | 35.00 | 3.55 | 5,161 | Dominated |  | 65,931 | 7 |
| SHWS, book | 34.56 | 3.55 | 4,712 | Dominated |  | 66,267 | 5 |
| GCBT, general | 34.49 | 3.55 | 5,409 | Dominated |  | 65,557 | 9 |
| SHNS, book | 34.23 | 3.54 | 4,472 |  |  | 66,424 | 4 |
| Exposure | 33.93 | 3.54 | 5,422 | Dominated |  | 65,403 | 11 |
| GCBT, Heimberg | 33.31 | 3.53 | 5,436 | Dominated |  | 65,232 | 13 |
| Paroxetine | 30.01 | 3.51 | 4,605 | Dominated |  | 65,561 | 8 |
| Venlafaxine | 29.45 | 3.50 | 4,689 | Dominated |  | 65,328 | 12 |
| SHNS, internet | 30.31 | 3.50 | 5,021 | Dominated |  | 64,898 | 18 |
| Fluvoxamine | 29.07 | 3.50 | 4,730 | Dominated |  | 65,185 | 15 |
| Sertraline | 28.74 | 3.49 | 4,626 | Dominated |  | 65,199 | 14 |
| PDPT | 29.53 | 3.49 | 6,716 | Dominated |  | 63,006 | 25 |
| Escitalopram | 28.11 | 3.48 | 4,632 | Dominated |  | 65,022 | 16 |
| Fluoxetine | 27.96 | 3.48 | 4,636 | Dominated |  | 64,980 | 17 |
| Citalopram | 27.52 | 3.47 | 4,641 | Dominated |  | 64,855 | 19 |
| Mirtazapine | 27.17 | 3.47 | 4,650 | Dominated |  | 64,751 | 20 |
| Moclobemide | 25.94 | 3.45 | 4,796 | Dominated |  | 64,271 | 21 |
| Pregabalin | 25.77 | 3.45 | 4,987 | Dominated |  | 64,034 | 22 |
| IPT | 26.01 | 3.44 | 6,154 | Dominated |  | 62,686 | 27 |
| Mindfulness | 25.40 | 3.43 | 5,021 | Dominated |  | 63,669 | 23 |
| Supportive therapy | 23.34 | 3.41 | 6,105 | Dominated |  | 62,066 | 28 |
| Pill placebo | 22.27 | 3.40 | 4,745 | Dominated |  | 63,335 | 24 |
| Wait list | 20.42 | 3.37 | 4,586 | Dominated |  | 62,826 | 26 |

1. **self-help interventions supported by a Band 5 therapist and group therapies delivered by one Band 7 and one Band 6 therapists**

| **Intervention** | **% without SA at 5 years** | **Mean QALYs** | **Mean total costs (£)** | **Incremental analysis & ICERs (£/QALY)** |  | **Mean NMB (£)** | **Ranking by highest NMB** |
| --- | --- | --- | --- | --- | --- | --- | --- |
| ICBT, C&W | 52.05 | 3.77 | 6,217 | 8,084 versus phenelzine |  | 69,144 | 1 |
| ICBT, general | 42.74 | 3.65 | 5,880 | Extendedly dominated^a^ |  | 67,150 | 2 |
| ICBT, Hope | 38.67 | 3.60 | 6,029 | Dominated |  | 65,982 | 7 |
| ICBT, short | 37.34 | 3.58 | 6,169 | Dominated |  | 65,509 | 10 |
| Phenelzine | 34.79 | 3.57 | 4,639 | 7,457 versus SHNS book |  | 66,819 | 3 |
| SHWS, internet | 35.00 | 3.55 | 5,039 | Dominated |  | 66,053 | 6 |
| SHWS, book | 34.56 | 3.55 | 4,590 | Dominated |  | 66,389 | 5 |
| GCBT, general | 34.49 | 3.55 | 5,322 | Dominated |  | 65,644 | 8 |
| SHNS, book | 34.23 | 3.54 | 4,429 |  |  | 66,468 | 4 |
| Exposure | 33.93 | 3.54 | 5,334 | Dominated |  | 65,490 | 11 |
| GCBT, Heimberg | 33.31 | 3.53 | 5,349 | Dominated |  | 65,319 | 13 |
| Paroxetine | 30.01 | 3.51 | 4,605 | Dominated |  | 65,561 | 9 |
| Venlafaxine | 29.45 | 3.50 | 4,689 | Dominated |  | 65,328 | 12 |
| SHNS, internet | 30.31 | 3.50 | 4,977 | Dominated |  | 64,941 | 18 |
| Fluvoxamine | 29.07 | 3.50 | 4,730 | Dominated |  | 65,185 | 15 |
| Sertraline | 28.74 | 3.49 | 4,626 | Dominated |  | 65,199 | 14 |
| PDPT | 29.53 | 3.49 | 6,716 | Dominated |  | 63,006 | 25 |
| Escitalopram | 28.11 | 3.48 | 4,632 | Dominated |  | 65,022 | 16 |
| Fluoxetine | 27.96 | 3.48 | 4,636 | Dominated |  | 64,980 | 17 |
| Citalopram | 27.52 | 3.47 | 4,641 | Dominated |  | 64,855 | 19 |
| Mirtazapine | 27.17 | 3.47 | 4,650 | Dominated |  | 64,751 | 20 |
| Moclobemide | 25.94 | 3.45 | 4,796 | Dominated |  | 64,271 | 21 |
| Pregabalin | 25.77 | 3.45 | 4,987 | Dominated |  | 64,034 | 22 |
| IPT | 26.01 | 3.44 | 6,154 | Dominated |  | 62,686 | 27 |
| Mindfulness | 25.40 | 3.43 | 4,981 | Dominated |  | 63,709 | 23 |
| Supportive therapy | 23.34 | 3.41 | 6,105 | Dominated |  | 62,066 | 28 |
| Pill placebo | 22.27 | 3.40 | 4,745 | Dominated |  | 63,335 | 24 |
| Wait list | 20.42 | 3.37 | 4,586 | Dominated |  | 62,826 | 26 |

1. **use of an alternative set of utility scores [40]**

| **Intervention** | **% without SA at 5 years** | **Mean QALYs** | **Mean total costs (£)** | **Incremental analysis & ICERs (£/QALY)** |  | **Mean NMB (£)** | **Ranking by highest NMB** |
| --- | --- | --- | --- | --- | --- | --- | --- |
| ICBT, C&W | 52.05 | 4.19 | 6,217 | 13,945 versus phenelzine |  | 77,483 | 1 |
| ICBT, general | 42.74 | 4.12 | 5,880 | Extendedly dominated^a^ |  | 76,469 | 4 |
| ICBT, Hope | 38.67 | 4.09 | 6,029 | Dominated |  | 75,729 | 14 |
| ICBT, short | 37.34 | 4.08 | 6,169 | Dominated |  | 75,396 | 20 |
| Phenelzine | 34.79 | 4.07 | 4,639 | 10,315 versus SHNS, book |  | 76,798 | 2 |
| SHWS, internet | 35.00 | 4.06 | 5,161 | Dominated |  | 76,064 | 7 |
| SHWS, book | 34.56 | 4.06 | 4,712 | Dominated |  | 76,447 | 5 |
| GCBT, general | 34.49 | 4.06 | 5,409 | Dominated |  | 75,743 | 12 |
| SHNS, book | 34.23 | 4.06 | 4,472 |  |  | 76,639 | 3 |
| Exposure | 33.93 | 4.05 | 5,422 | Dominated |  | 75,648 | 16 |
| GCBT, Heimberg | 33.31 | 4.05 | 5,436 | Dominated |  | 75,543 | 18 |
| Paroxetine | 30.01 | 4.03 | 4,605 | Dominated |  | 76,084 | 6 |
| Venlafaxine | 29.45 | 4.03 | 4,689 | Dominated |  | 75,913 | 8 |
| SHNS, internet | 30.31 | 4.03 | 5,021 | Dominated |  | 75,524 | 19 |
| Fluvoxamine | 29.07 | 4.03 | 4,730 | Dominated |  | 75,813 | 10 |
| Sertraline | 28.74 | 4.02 | 4,626 | Dominated |  | 75,865 | 9 |
| PDPT | 29.53 | 4.02 | 6,716 | Dominated |  | 73,715 | 27 |
| Escitalopram | 28.11 | 4.02 | 4,632 | Dominated |  | 75,759 | 11 |
| Fluoxetine | 27.96 | 4.02 | 4,636 | Dominated |  | 75,733 | 13 |
| Citalopram | 27.52 | 4.01 | 4,641 | Dominated |  | 75,659 | 15 |
| Mirtazapine | 27.17 | 4.01 | 4,650 | Dominated |  | 75,595 | 17 |
| Moclobemide | 25.94 | 4.00 | 4,796 | Dominated |  | 75,255 | 21 |
| Pregabalin | 25.77 | 4.00 | 4,987 | Dominated |  | 75,038 | 22 |
| IPT | 26.01 | 4.00 | 6,154 | Dominated |  | 73,766 | 26 |
| Mindfulness | 25.40 | 3.99 | 5,021 | Dominated |  | 74,812 | 23 |
| Supportive therapy | 23.34 | 3.98 | 6,105 | Dominated |  | 73,427 | 28 |
| Pill placebo | 22.27 | 3.97 | 4,745 | Dominated |  | 74,734 | 24 |
| Wait list | 20.42 | 3.95 | 4,586 | Dominated |  | 74,506 | 25 |

**d1. Time horizon of 1 year + 12 weeks of initial treatment**

| **Intervention** | **% without SA at 5 years** | **Mean QALYs** | **Mean total costs (£)** | **Incremental analysis & ICERs (£/QALY)** |  | **Mean NMB (£)** | **Ranking by highest NMB** |
| --- | --- | --- | --- | --- | --- | --- | --- |
| ICBT, C&W | 53.63 | 0.95 | 3,169 | 47,687 versus phenelzine |  | 15,750 | 17 |
| ICBT, general | 40.72 | 0.91 | 2,673 | Extendedly dominated^a^ |  | 15,596 | 20 |
| Phenelzine | 29.69 | 0.91 | 1,296 | 7,725 versus SHNS, book |  | 16,837 | 1 |
| ICBT Hope | 35.07 | 0.90 | 2,752 | Dominated |  | 15,233 | 24 |
| ICBT, short | 33.23 | 0.89 | 2,870 | Dominated |  | 15,023 | 25 |
| SHWS, internet | 29.98 | 0.89 | 1,821 | Dominated |  | 15,908 | 13 |
| Paroxetine | 23.05 | 0.89 | 1,180 | Extendedly dominated^a^ |  | 16,525 | 3 |
| SHWS, book | 29.36 | 0.88 | 1,365 | Dominated |  | 16,333 | 8 |
| GCBT, general | 29.28 | 0.88 | 2,061 | Dominated |  | 15,633 | 19 |
| SHNS, book | 28.91 | 0.88 | 1,119 | 2,453 versus wait list |  | 16,556 | 2 |
| Venlafaxine | 22.28 | 0.88 | 1,255 | Dominated |  | 16,402 | 5 |
| Exposure | 28.49 | 0.88 | 2,064 | Dominated |  | 15,591 | 21 |
| Fluvoxamine | 21.76 | 0.88 | 1,290 | Dominated |  | 16,333 | 9 |
| GCBT, Heimberg | 27.63 | 0.88 | 2,068 | Dominated |  | 15,543 | 22 |
| Sertraline | 21.29 | 0.88 | 1,180 | Dominated |  | 16,413 | 4 |
| Escitalopram | 20.42 | 0.88 | 1,175 | Dominated |  | 16,361 | 6 |
| Fluoxetine | 20.22 | 0.88 | 1,176 | Dominated |  | 16,347 | 7 |
| Citalopram | 19.60 | 0.87 | 1,173 | Dominated |  | 16,311 | 10 |
| Mirtazapine | 19.11 | 0.87 | 1,176 | Dominated |  | 16,276 | 11 |
| SHNS, internet | 23.48 | 0.87 | 1,601 | Dominated |  | 15,801 | 16 |
| PDPT | 22.38 | 0.87 | 3,283 | Dominated |  | 14,065 | 28 |
| Moclobemide | 17.40 | 0.87 | 1,302 | Dominated |  | 16,040 | 12 |
| Pregabalin | 17.17 | 0.87 | 1,490 | Dominated |  | 15,837 | 14 |
| IPT | 17.50 | 0.86 | 2,661 | Dominated |  | 14,441 | 26 |
| Mindfulness | 16.67 | 0.85 | 1,518 | Dominated |  | 15,542 | 23 |
| Placebo | 12.33 | 0.85 | 1,188 | Dominated |  | 15,827 | 15 |
| Supportive therapy | 13.80 | 0.85 | 2,567 | Dominated |  | 14,349 | 27 |
| Wait list | 9.76 | 0.83 | 998 |  |  | 15,685 | 18 |

**d2. Time horizon of 3 years + 12 weeks of initial treatment**

| **Intervention** | **% without SA at 5 years** | **Mean QALYs** | **Mean total costs (£)** | **Incremental analysis & ICERs (£/QALY)** |  | **Mean NMB (£)** | **Ranking by highest NMB** |
| --- | --- | --- | --- | --- | --- | --- | --- |
| ICBT, C&W | 52.77 | 2.41 | 4,743 | 13,531 versus phenelzine |  | 43,398 | 1 |
| ICBT, general | 41.81 | 2.33 | 4,335 | Extendedly dominated^a^ |  | 42,218 | 4 |
| ICBT Hope | 37.01 | 2.29 | 4,453 | Dominated |  | 41,405 | 11 |
| ICBT, short | 35.45 | 2.28 | 4,584 | Dominated |  | 41,049 | 20 |
| Phenelzine | 32.45 | 2.28 | 3,034 | 6,667 versus SHNS. book |  | 42,581 | 2 |
| SHWS, internet | 32.69 | 2.26 | 3,558 | Dominated |  | 41,675 | 7 |
| SHWS, book | 32.17 | 2.26 | 3,106 | Dominated |  | 42,052 | 5 |
| GCBT, general | 32.10 | 2.26 | 3,802 | Dominated |  | 41,345 | 13 |
| SHNS, book | 31.78 | 2.26 | 2,863 |  |  | 42,238 | 3 |
| Exposure | 31.43 | 2.25 | 3,810 | Dominated |  | 41,240 | 16 |
| GCBT, Heimberg | 30.70 | 2.25 | 3,820 | Dominated |  | 41,124 | 18 |
| Paroxetine | 26.81 | 2.24 | 2,964 | Dominated |  | 41,742 | 6 |
| Venlafaxine | 26.16 | 2.23 | 3,044 | Dominated |  | 41,557 | 8 |
| Fluvoxamine | 25.72 | 2.23 | 3,082 | Dominated |  | 41,447 | 10 |
| Sertraline | 25.32 | 2.22 | 2,975 | Dominated |  | 41,490 | 9 |
| SHNS, internet | 27.17 | 2.22 | 3,382 | Dominated |  | 41,051 | 19 |
| Escitalopram | 24.58 | 2.22 | 2,977 | Dominated |  | 41,368 | 12 |
| Fluoxetine | 24.41 | 2.22 | 2,979 | Dominated |  | 41,339 | 14 |
| PDPT | 26.25 | 2.21 | 5,071 | Dominated |  | 39,228 | 26 |
| Citalopram | 23.89 | 2.21 | 2,981 | Dominated |  | 41,253 | 15 |
| Mirtazapine | 23.47 | 2.21 | 2,987 | Dominated |  | 41,180 | 17 |
| Moclobemide | 22.02 | 2.20 | 3,125 | Dominated |  | 40,808 | 21 |
| Pregabalin | 21.82 | 2.20 | 3,314 | Dominated |  | 40,586 | 22 |
| IPT | 22.10 | 2.18 | 4,483 | Dominated |  | 39,216 | 27 |
| Mindfulness | 21.39 | 2.18 | 3,345 | Dominated |  | 40,251 | 23 |
| Supportive therapy | 18.96 | 2.16 | 4,414 | Dominated |  | 38,830 | 28 |
| Placebo | 17.71 | 2.16 | 3,045 | Dominated |  | 40,191 | 24 |
| Wait list | 15.53 | 2.14 | 2,873 | Dominated |  | 39,845 | 25 |

**d3. Time horizon of 10 years + 12 weeks of initial treatment**

| **Intervention** | **% without SA at 5 years** | **Mean QALYs** | **Mean total costs (£)** | **Incremental analysis & ICERs (£/QALY)** |  | **Mean NMB (£)** | **Ranking by highest NMB** |
| --- | --- | --- | --- | --- | --- | --- | --- |
| ICBT, C&W | 52.05 | 6.78 | 7,147 | Dominant |  | 128,435 | 1 |
| ICBT, general | 42.74 | 6.60 | 7,479 | Dominated |  | 124,520 | 2 |
| ICBT Hope | 38.67 | 6.52 | 7,624 | Dominated |  | 122,807 | 3 |
| ICBT, short | 37.34 | 6.50 | 7,671 | Dominated |  | 122,249 | 4 |
| Phenelzine | 34.79 | 6.47 | 7,730 | Dominated |  | 121,627 | 5 |
| SHWS, internet | 35.00 | 6.45 | 7,755 | Dominated |  | 121,264 | 6 |
| SHWS, book | 34.56 | 6.44 | 7,771 | Dominated |  | 121,079 | 7 |
| GCBT, general | 34.49 | 6.44 | 7,773 | Dominated |  | 121,052 | 8 |
| SHNS, book | 34.23 | 6.44 | 7,783 | Dominated |  | 120,939 | 9 |
| Exposure | 33.93 | 6.43 | 7,793 | Dominated |  | 120,815 | 10 |
| GCBT, Heimberg | 33.31 | 6.42 | 7,815 | Dominated |  | 120,552 | 11 |
| Paroxetine | 30.01 | 6.37 | 7,908 | Dominated |  | 119,514 | 12 |
| SHNS, internet | 30.31 | 6.36 | 7,922 | Dominated |  | 119,293 | 13 |
| Venlafaxine | 29.45 | 6.36 | 7,929 | Dominated |  | 119,268 | 14 |
| Fluvoxamine | 29.07 | 6.35 | 7,943 | Dominated |  | 119,103 | 15 |
| PDPT | 29.53 | 6.35 | 7,950 | Dominated |  | 118,962 | 16 |
| Sertraline | 28.74 | 6.35 | 7,955 | Dominated |  | 118,955 | 17 |
| Escitalopram | 28.11 | 6.33 | 7,979 | Dominated |  | 118,676 | 18 |
| Fluoxetine | 27.96 | 6.33 | 7,984 | Dominated |  | 118,612 | 19 |
| Citalopram | 27.52 | 6.32 | 8,001 | Dominated |  | 118,417 | 20 |
| Mirtazapine | 27.17 | 6.31 | 8,014 | Dominated |  | 118,261 | 21 |
| Moclobemide | 25.94 | 6.29 | 8,059 | Dominated |  | 117,716 | 22 |
| Pregabalin | 25.77 | 6.29 | 8,066 | Dominated |  | 117,641 | 23 |
| IPT | 26.01 | 6.28 | 8,076 | Dominated |  | 117,482 | 24 |
| Mindfulness | 25.40 | 6.27 | 8,097 | Dominated |  | 117,229 | 25 |
| Supportive therapy | 23.34 | 6.23 | 8,171 | Dominated |  | 116,359 | 26 |
| Placebo | 22.27 | 6.21 | 8,196 | Dominated |  | 116,100 | 27 |
| Wait list | 20.42 | 6.17 | 8,277 | Dominated |  | 115,102 | 28 |

**e1. Risk ratio of relapse of drugs versus psychological interventions (year 1) set at 1.00**

| **Intervention** | **% without SA at 5 years** | **Mean QALYs** | **Mean total costs (£)** | **Incremental analysis & ICERs (£/QALY)** |  | **Mean NMB (£)** | **Ranking by highest NMB** |
| --- | --- | --- | --- | --- | --- | --- | --- |
| ICBT, C&W | 39.57 | 3.64 | 6,464 | 28,281 versus phenelzine |  | 66,284 | 2 |
| Phenelzine | 34.79 | 3.57 | 4,639 | 529 versus paroxetine |  | 66,819 | 1 |
| ICBT, general | 33.27 | 3.55 | 6,068 | Dominated |  | 64,979 | 9 |
| ICBT Hope | 30.51 | 3.52 | 6,190 | Dominated |  | 64,112 | 16 |
| Paroxetine | 30.01 | 3.51 | 4,605 | 138 versus wait list |  | 65,561 | 3 |
| ICBT, short | 29.61 | 3.50 | 6,322 | Dominated |  | 63,737 | 21 |
| Venlafaxine | 29.45 | 3.50 | 4,689 | Dominated |  | 65,328 | 4 |
| Fluvoxamine | 29.07 | 3.50 | 4,730 | Dominated |  | 65,185 | 6 |
| Sertraline | 28.74 | 3.49 | 4,626 | Dominated |  | 65,199 | 5 |
| Escitalopram | 28.11 | 3.48 | 4,632 | Dominated |  | 65,022 | 7 |
| SHWS, internet | 28.02 | 3.48 | 5,299 | Dominated |  | 64,333 | 14 |
| Fluoxetine | 27.96 | 3.48 | 4,636 | Dominated |  | 64,980 | 8 |
| SHWS, book | 27.72 | 3.48 | 4,848 | Dominated |  | 64,701 | 13 |
| GCBT, general | 27.68 | 3.48 | 5,544 | Dominated |  | 63,995 | 18 |
| Citalopram | 27.52 | 3.47 | 4,641 | Dominated |  | 64,855 | 11 |
| SHNS, book | 27.50 | 3.47 | 4,606 | Dominated |  | 64,883 | 10 |
| Exposure | 27.30 | 3.47 | 5,553 | Dominated |  | 63,883 | 19 |
| Mirtazapine | 27.17 | 3.47 | 4,650 | Dominated |  | 64,751 | 12 |
| GCBT, Heimberg | 26.88 | 3.47 | 5,563 | Dominated |  | 63,758 | 20 |
| Moclobemide | 25.94 | 3.45 | 4,796 | Dominated |  | 64,271 | 15 |
| Pregabalin | 25.77 | 3.45 | 4,987 | Dominated |  | 64,034 | 17 |
| SHNS, internet | 24.85 | 3.44 | 5,129 | Dominated |  | 63,646 | 22 |
| PDPT | 24.32 | 3.43 | 6,819 | Dominated |  | 61,812 | 26 |
| Placebo | 22.27 | 3.40 | 4,745 | Dominated |  | 63,335 | 23 |
| IPT | 21.93 | 3.40 | 6,235 | Dominated |  | 61,752 | 27 |
| Mindfulness | 21.53 | 3.39 | 5,098 | Dominated |  | 62,780 | 25 |
| Supportive therapy | 20.13 | 3.37 | 6,169 | Dominated |  | 61,330 | 28 |
| Wait list | 20.42 | 3.37 | 4,586 |  |  | 62,826 | 24 |

**e2. Risk ratio of relapse of drugs versus psychological interventions (year 1) set at 1.25**

| **Intervention** | **% without SA at 5 years** | **Mean QALYs** | **Mean total costs (£)** | **Incremental analysis & ICERs (£/QALY)** |  | **Mean NMB (£)** | **Ranking by highest NMB** |
| --- | --- | --- | --- | --- | --- | --- | --- |
| ICBT, C&W | 43.31 | 3.68 | 6,390 | 16,880 versus phenelzine |  | 67,142 | 1 |
| ICBT, general | 36.11 | 3.58 | 6,011 | Extendedly dominated^a^ |  | 65,630 | 3 |
| Phenelzine | 34.79 | 3.57 | 4,639 | 929 versus SHNS, book |  | 66,819 | 2 |
| ICBT Hope | 32.96 | 3.54 | 6,142 | Dominated |  | 64,673 | 15 |
| ICBT, short | 31.93 | 3.53 | 6,276 | Dominated |  | 64,269 | 19 |
| Paroxetine | 30.01 | 3.51 | 4,605 | Extendedly dominated^a^ |  | 65,561 | 4 |
| SHWS, internet | 30.12 | 3.50 | 5,258 | Dominated |  | 64,812 | 13 |
| Venlafaxine | 29.45 | 3.50 | 4,689 | Dominated |  | 65,328 | 6 |
| SHWS, book | 29.77 | 3.50 | 4,807 | Dominated |  | 65,171 | 9 |
| GCBT, general | 29.73 | 3.50 | 5,503 | Dominated |  | 64,464 | 16 |
| Fluvoxamine | 29.07 | 3.50 | 4,730 | Dominated |  | 65,185 | 8 |
| SHNS, book | 29.52 | 3.50 | 4,566 |  |  | 65,345 | 5 |
| Exposure | 29.29 | 3.49 | 5,514 | Dominated |  | 64,339 | 17 |
| Sertraline | 28.74 | 3.49 | 4,626 | Dominated |  | 65,199 | 7 |
| GCBT, Heimberg | 28.81 | 3.49 | 5,525 | Dominated |  | 64,200 | 20 |
| Escitalopram | 28.11 | 3.48 | 4,632 | Dominated |  | 65,022 | 10 |
| Fluoxetine | 27.96 | 3.48 | 4,636 | Dominated |  | 64,980 | 11 |
| Citalopram | 27.52 | 3.47 | 4,641 | Dominated |  | 64,855 | 12 |
| Mirtazapine | 27.17 | 3.47 | 4,650 | Dominated |  | 64,751 | 14 |
| SHNS, internet | 26.49 | 3.46 | 5,096 | Dominated |  | 64,021 | 22 |
| Moclobemide | 25.94 | 3.45 | 4,796 | Dominated |  | 64,271 | 18 |
| Pregabalin | 25.77 | 3.45 | 4,987 | Dominated |  | 64,034 | 21 |
| PDPT | 25.88 | 3.45 | 6,788 | Dominated |  | 62,170 | 26 |
| IPT | 23.15 | 3.41 | 6,211 | Dominated |  | 62,032 | 27 |
| Mindfulness | 22.69 | 3.41 | 5,075 | Dominated |  | 63,046 | 24 |
| Placebo | 22.27 | 3.40 | 4,745 | Dominated |  | 63,335 | 23 |
| Supportive therapy | 21.09 | 3.39 | 6,150 | Dominated |  | 61,551 | 28 |
| Wait list | 20.42 | 3.37 | 4,586 | Dominated |  | 62,826 | 25 |

**e3. Risk ratio of relapse of drugs versus psychological interventions (year 1) set at 1.50**

| **Intervention** | **% without SA at 5 years** | **Mean QALYs** | **Mean total costs (£)** | **Incremental analysis & ICERs (£/QALY)** |  | **Mean NMB (£)** | **Ranking by highest NMB** |
| --- | --- | --- | --- | --- | --- | --- | --- |
| ICBT, C&W | 45.81 | 3.70 | 6,341 | 13,102 versus phenelzine |  | 67,714 | 1 |
| ICBT, general | 38.00 | 3.60 | 5,974 | Extendedly dominated^a^ |  | 66,065 | 3 |
| Phenelzine | 34.79 | 3.57 | 4,639 | 1,557 versus SHNS, book |  | 66,819 | 2 |
| ICBT Hope | 34.59 | 3.56 | 6,109 | Dominated |  | 65,047 | 11 |
| ICBT, short | 33.48 | 3.54 | 6,246 | Dominated |  | 64,623 | 18 |
| SHWS, internet | 31.51 | 3.52 | 5,230 | Dominated |  | 65,132 | 10 |
| SHWS, book | 31.14 | 3.51 | 4,780 | Dominated |  | 65,484 | 6 |
| GCBT, general | 31.09 | 3.51 | 5,476 | Dominated |  | 64,776 | 15 |
| SHNS, book | 30.86 | 3.51 | 4,539 |  |  | 65,653 | 4 |
| Paroxetine | 30.01 | 3.51 | 4,605 | Dominated |  | 65,561 | 5 |
| Exposure | 30.62 | 3.51 | 5,487 | Dominated |  | 64,643 | 17 |
| Venlafaxine | 29.45 | 3.50 | 4,689 | Dominated |  | 65,328 | 7 |
| GCBT, Heimberg | 30.09 | 3.50 | 5,500 | Dominated |  | 64,495 | 19 |
| Fluvoxamine | 29.07 | 3.50 | 4,730 | Dominated |  | 65,185 | 9 |
| Sertraline | 28.74 | 3.49 | 4,626 | Dominated |  | 65,199 | 8 |
| Escitalopram | 28.11 | 3.48 | 4,632 | Dominated |  | 65,022 | 12 |
| Fluoxetine | 27.96 | 3.48 | 4,636 | Dominated |  | 64,980 | 13 |
| Citalopram | 27.52 | 3.47 | 4,641 | Dominated |  | 64,855 | 14 |
| Mirtazapine | 27.17 | 3.47 | 4,650 | Dominated |  | 64,751 | 16 |
| SHNS, internet | 27.58 | 3.47 | 5,075 | Dominated |  | 64,272 | 20 |
| PDPT | 26.92 | 3.46 | 6,767 | Dominated |  | 62,409 | 26 |
| Moclobemide | 25.94 | 3.45 | 4,796 | Dominated |  | 64,271 | 21 |
| Pregabalin | 25.77 | 3.45 | 4,987 | Dominated |  | 64,034 | 22 |
| IPT | 23.97 | 3.42 | 6,195 | Dominated |  | 62,219 | 27 |
| Mindfulness | 23.47 | 3.41 | 5,059 | Dominated |  | 63,224 | 24 |
| Placebo | 22.27 | 3.40 | 4,745 | Dominated |  | 63,335 | 23 |
| Supportive therapy | 21.73 | 3.39 | 6,137 | Dominated |  | 61,698 | 28 |
| Wait list | 20.42 | 3.37 | 4,586 | Dominated |  | 62,826 | 25 |

**e4. Risk ratio of relapse of drugs versus psychological interventions (year 1) set at 1.75**

| **Intervention** | **% without SA at 5 years** | **Mean QALYs** | **Mean total costs (£)** | **Incremental analysis & ICERs (£/QALY)** |  | **Mean NMB (£)** | **Ranking by highest NMB** |
| --- | --- | --- | --- | --- | --- | --- | --- |
| ICBT, C&W | 47.59 | 3.72 | 6,305 | 11,218 versus phenelzine |  | 68,123 | 1 |
| ICBT, general | 39.36 | 3.62 | 5,947 | Extendedly dominated^a^ |  | 66,375 | 3 |
| Phenelzine | 34.79 | 3.57 | 4,639 | 2,210 versus SHNS, book |  | 66,819 | 2 |
| ICBT Hope | 35.75 | 3.57 | 6,086 | Dominated |  | 65,314 | 9 |
| ICBT, short | 34.58 | 3.56 | 6,224 | Dominated |  | 64,876 | 15 |
| SHWS, internet | 32.51 | 3.53 | 5,210 | Dominated |  | 65,360 | 7 |
| SHWS, book | 32.12 | 3.52 | 4,761 | Dominated |  | 65,708 | 5 |
| GCBT, general | 32.06 | 3.52 | 5,457 | Dominated |  | 64,999 | 13 |
| SHNS, book | 31.82 | 3.52 | 4,520 |  |  | 65,874 | 4 |
| Exposure | 31.56 | 3.52 | 5,469 | Dominated |  | 64,860 | 16 |
| GCBT, Heimberg | 31.01 | 3.51 | 5,482 | Dominated |  | 64,706 | 19 |
| Paroxetine | 30.01 | 3.51 | 4,605 | Dominated |  | 65,561 | 6 |
| Venlafaxine | 29.45 | 3.50 | 4,689 | Dominated |  | 65,328 | 8 |
| Fluvoxamine | 29.07 | 3.50 | 4,730 | Dominated |  | 65,185 | 11 |
| Sertraline | 28.74 | 3.49 | 4,626 | Dominated |  | 65,199 | 10 |
| Escitalopram | 28.11 | 3.48 | 4,632 | Dominated |  | 65,022 | 12 |
| Fluoxetine | 27.96 | 3.48 | 4,636 | Dominated |  | 64,980 | 14 |
| SHNS, internet | 28.36 | 3.48 | 5,059 | Dominated |  | 64,450 | 20 |
| Citalopram | 27.52 | 3.47 | 4,641 | Dominated |  | 64,855 | 17 |
| Mirtazapine | 27.17 | 3.47 | 4,650 | Dominated |  | 64,751 | 18 |
| PDPT | 27.66 | 3.47 | 6,753 | Dominated |  | 62,579 | 26 |
| Moclobemide | 25.94 | 3.45 | 4,796 | Dominated |  | 64,271 | 21 |
| Pregabalin | 25.77 | 3.45 | 4,987 | Dominated |  | 64,034 | 22 |
| IPT | 24.55 | 3.43 | 6,183 | Dominated |  | 62,352 | 27 |
| Mindfulness | 24.02 | 3.42 | 5,048 | Dominated |  | 63,351 | 23 |
| Placebo | 22.27 | 3.40 | 4,745 | Dominated |  | 63,335 | 24 |
| Supportive therapy | 22.19 | 3.40 | 6,128 | Dominated |  | 61,803 | 28 |
| Wait list | 20.42 | 3.37 | 4,586 | Dominated |  | 62,826 | 25 |

**e5. Risk ratio of relapse of drugs versus psychological interventions (year 1) set at 2.00**

| **Intervention** | **% without SA at 5 years** | **Mean QALYs** | **Mean total costs (£)** | **Incremental analysis & ICERs (£/QALY)** |  | **Mean NMB (£)** | **Ranking by highest NMB** |
| --- | --- | --- | --- | --- | --- | --- | --- |
| ICBT, C&W | 48.93 | 3.74 | 6,279 | 10,089 versus phenelzine |  | 68,429 | 1 |
| ICBT, general | 40.37 | 3.63 | 5,927 | Extendedly dominated^a^ |  | 66,607 | 3 |
| ICBT Hope | 36.63 | 3.58 | 6,069 | Dominated |  | 65,514 | 8 |
| Phenelzine | 34.79 | 3.57 | 4,639 | 2,889 versus SHNS, book |  | 66,819 | 2 |
| ICBT, short | 35.41 | 3.56 | 6,208 | Dominated |  | 65,066 | 13 |
| SHWS, internet | 33.26 | 3.54 | 5,195 | Dominated |  | 65,531 | 7 |
| SHWS, book | 32.85 | 3.53 | 4,746 | Dominated |  | 65,875 | 5 |
| GCBT, general | 32.79 | 3.53 | 5,443 | Dominated |  | 65,166 | 12 |
| SHNS, book | 32.55 | 3.53 | 4,506 |  |  | 66,039 | 4 |
| Exposure | 32.27 | 3.52 | 5,455 | Dominated |  | 65,023 | 14 |
| GCBT, Heimberg | 31.70 | 3.52 | 5,468 | Dominated |  | 64,863 | 17 |
| Paroxetine | 30.01 | 3.51 | 4,605 | Dominated |  | 65,561 | 6 |
| Venlafaxine | 29.45 | 3.50 | 4,689 | Dominated |  | 65,328 | 9 |
| Fluvoxamine | 29.07 | 3.50 | 4,730 | Dominated |  | 65,185 | 11 |
| Sertraline | 28.74 | 3.49 | 4,626 | Dominated |  | 65,199 | 10 |
| Escitalopram | 28.11 | 3.48 | 4,632 | Dominated |  | 65,022 | 15 |
| SHNS, internet | 28.95 | 3.48 | 5,048 | Dominated |  | 64,585 | 20 |
| Fluoxetine | 27.96 | 3.48 | 4,636 | Dominated |  | 64,980 | 16 |
| Citalopram | 27.52 | 3.47 | 4,641 | Dominated |  | 64,855 | 18 |
| PDPT | 28.22 | 3.47 | 6,741 | Dominated |  | 62,707 | 26 |
| Mirtazapine | 27.17 | 3.47 | 4,650 | Dominated |  | 64,751 | 19 |
| Moclobemide | 25.94 | 3.45 | 4,796 | Dominated |  | 64,271 | 21 |
| Pregabalin | 25.77 | 3.45 | 4,987 | Dominated |  | 64,034 | 22 |
| IPT | 24.99 | 3.43 | 6,174 | Dominated |  | 62,452 | 27 |
| Mindfulness | 24.44 | 3.42 | 5,040 | Dominated |  | 63,446 | 23 |
| Placebo | 22.27 | 3.40 | 4,745 | Dominated |  | 63,335 | 24 |
| Supportive therapy | 22.53 | 3.40 | 6,121 | Dominated |  | 61,882 | 28 |
| Wait list | 20.42 | 3.37 | 4,586 | Dominated |  | 62,826 | 25 |

Notes for all tables in S4 Appendix:

^a^Extended dominance occurs when an option is less effective and more costly than a linear combination of two alternative options

Interventions have been ranked from the most to least effective according to the number of QALYs gained.

C&W: Clark and Wells model; GCBT: group cognitive behavioural therapy; ICBT: individually delivered cognitive behavioural therapy; ICER: Incremental Cost Effectiveness Ratio; IPT: interpersonal therapy; NMB: Net Monetary Benefit, estimated using a willingeness to pay £20,000/QALY; PDPT: psychodynamic psychotherapy; SA: Social Anxiety disorder; SHNS: self-help no support; SHWS: self-help with support.
