# Supplementary material for: The Cost Effectiveness of Psychological and Pharmacological Interventions for Social Anxiety Disorder: A Model-Based Economic Analysis
Source: PLoS One. 2015 Oct 27;10(10):e0140704. doi: 10.1371/journal.pone.0140704 (PMC4624770; doi:10.1371/journal.pone.0140704)
Supplement: S1 Table — Mean values (95% credible intervals) per person, 5 years after end of treatment (DOCX) [file pone.0140704.s004.docx]

**Detailed results of probabilistic sensitivity analysis.** Mean values (95% credible intervals) per person, 5 years after end of treatment

| **Intervention** | **Mean QALYs**  **(95% CIs)** | **Mean total costs (£)**  **(95% CIs)** | **Mean NMB (£)**  **(95% CIs)** | **Mean ranking by highest NMB** | **Probability of being best*** |
| --- | --- | --- | --- | --- | --- |
| ICBT, C&W | 3.75 (3.32 to 4.06) | 6,178 (5,035 to 7,705) | 68,810 (59,451 to 75,703) | 2.59 | 0.68 |
| ICBT, general | 3.64 (3.22 to 4.01) | 5,714 (4,097 to 7,447) | 67,040 (58,059 to 75,170) | 6.14 | 0.07 |
| ICBT, Hope | 3.59 (3.17 to 3.98) | 5,853 (4,128 to 7,611) | 65,916 (57,100 to 74,501) | 11.32 | 0.03 |
| ICBT, short | 3.57 (3.17 to 3.96) | 5,970 (4,197 to 7,789) | 65,479 (56,910 to 73,967) | 13.16 | 0.00 |
| Phenelzine | 3.57 (3.20 to 3.87) | 4,557 (3,328 to 6,136) | 66,899 (59,062 to 73,191) | 4.29 | 0.15 |
| SHWS, internet | 3.54 (3.16 to 3.94) | 5,186 (3,870 to 6,874) | 65,699 (57,411 to 74,294) | 10.82 | 0.00 |
| SHWS, book | 3.54 (3.15 to 3.94) | 4,741 (3,426 to 6,475) | 66,037 (57,731 to 74,698) | 9.03 | 0.02 |
| GCBT, general | 3.54 (3.15 to 3.93) | 5,436 (4,144 to 7,130) | 65,327 (57,013 to 73,908) | 13.54 | 0.00 |
| SHNS, book | 3.53 (3.15 to 3.93) | 4,501 (3,206 to 6,198) | 66,197 (57,903 to 74,793) | 7.37 | 0.02 |
| Exposure | 3.53 (3.14 to 3.93) | 5,448 (4,148 to 7,155) | 65,179 (56,906 to 73,817) | 14.47 | 0.00 |
| GCBT, Heimberg | 3.52 (3.14 to 3.92) | 5,463 (4,149 to 7,171) | 65,013 (56,830 to 73,653) | 15.63 | 0.00 |
| Paroxetine | 3.51 (3.15 to 3.83) | 4,561 (3,255 to 6,237) | 65,603 (57,963 to 72,440) | 9.24 | 0.00 |
| Venlafaxine | 3.50 (3.14 to 3.82) | 4,633 (3,294 to 6,300) | 65,380 (57,807 to 72,279) | 10.98 | 0.00 |
| Fluvoxamine | 3.50 (3.13 to 3.82) | 4,666 (3,329 to 6,353) | 65,245 (57,604 to 72,135) | 11.98 | 0.00 |
| Sertraline | 3.49 (3.13 to 3.82) | 4,583 (3,242 to 6,267) | 65,239 (57,648 to 72,161) | 11.94 | 0.00 |
| SHNS, internet | 3.49 (3.11 to 3.89) | 5,042 (3,675 to 6,792) | 64,714 (56,768 to 73,395) | 16.76 | 0.00 |
| Escitalopram | 3.48 (3.12 to 3.81) | 4,591 (3,242 to 6,293) | 65,058 (57,494 to 72,114) | 13.18 | 0.00 |
| Fluoxetine | 3.48 (3.12 to 3.81) | 4,597 (3,241 to 6,311) | 65,016 (57,514 to 72,015) | 13.45 | 0.00 |
| PDPT | 3.48 (3.11 to 3.87) | 6,509 (4,494 to 8,414) | 63,055 (55,168 to 71,680) | 24.02 | 0.00 |
| Citalopram | 3.47 (3.11 to 3.80) | 4,602 (3,242 to 6,334) | 64,890 (57,279 to 71,920) | 14.21 | 0.01 |
| Mirtazapine | 3.47 (3.11 to 3.81) | 4,611 (3,248 to 6,345) | 64,786 (57,054 to 72,114) | 14.82 | 0.02 |
| Moclobemide | 3.45 (3.10 to 3.78) | 4,740 (3,352 to 6,472) | 64,323 (56,925 to 71,314) | 18.00 | 0.00 |
| Pregabalin | 3.45 (3.10 to 3.78) | 4,898 (3,478 to 6,622) | 64,118 (56,751 to 71,121) | 19.18 | 0.00 |
| IPT | 3.44 (3.08 to 3.82) | 5,987 (4,252 to 7,858) | 62,728 (55,144 to 71,060) | 24.94 | 0.00 |
| Mindfulness | 3.43 (3.07 to 3.80) | 5,041 (3,607 to 6,841) | 63,527 (56,022 to 71,746) | 22.03 | 0.00 |
| Pill placebo | 3.40 (3.06 to 3.73) | 4,713 (3,257 to 6,517) | 63,360 (56,308 to 70,281) | 22.54 | 0.00 |
| Supportive therapy | 3.40 (3.05 to 3.77) | 5,934 (4,189 to 7,830) | 62,136 (54,773 to 70,120) | 26.33 | 0.00 |
| Wait list | 3.37 (3.03 to 3.70) | 4,593 (3,087 to 6,442) | 62,810 (55,846 to 69,886) | 24.04 | 0.00 |

* at a cost effectiveness threshold of £20,000/QALY

Interventions have been ranked from the most to least effective according to the number of QALYs gained.

C&W: Clark and Wells model; CI: credible interval; GCBT: group cognitive behavioural therapy; ICBT: individually delivered cognitive behavioural therapy; IPT: interpersonal therapy; NMB: Net Monetary Benefit, estimated using a willingeness to pay £20,000/QALY; PDPT: psychodynamic psychotherapy; SHNS: self-help no support; SHWS: self-help with support.
