# Supplementary material for: The Cost Effectiveness of Psychological and Pharmacological Interventions for Social Anxiety Disorder: A Model-Based Economic Analysis
Source: PLoS One. 2015 Oct 27;10(10):e0140704. doi: 10.1371/journal.pone.0140704 (PMC4624770; doi:10.1371/journal.pone.0140704)
Supplement: S2 Table — Probability of cost effectiveness of the interventions with the highest net monetary benefit at varying levels of willingness-to-pay per QALY gained. (DOCX) [file pone.0140704.s005.docx]

**Values used to construct Figure 3.** Probability of cost effectiveness of the interventions with the highest net monetary benefit at varying levels of willingness-to-pay per QALY gained.

| **Probability of cost effectiveness of the intervention with the highest Net Monetary Benefit at each level of willingness-to-pay** | | | **Willingness-to-pay per QALY (£)** |
| --- | --- | --- | --- |
| **Book-based self help without support** | **Phenelzine** | **C&W Cognitive therapy** |  |
| 0.1802 |  |  | 0 |
| 0.2139 |  |  | 500 |
| 0.2304 |  |  | 1000 |
| 0.2362 | 0.2777 |  | 1472 |
|  | 0.2793 |  | 1500 |
|  | 0.3066 |  | 2000 |
|  | 0.3295 |  | 2500 |
|  | 0.3487 |  | 3000 |
|  | 0.3670 |  | 3500 |
|  | 0.3779 |  | 4000 |
|  | 0.3816 |  | 4500 |
|  | 0.3819 |  | 5000 |
|  | 0.3769 |  | 5500 |
|  | 0.3704 |  | 6000 |
|  | 0.3626 |  | 6500 |
|  | 0.3539 |  | 7000 |
|  | 0.3434 |  | 7500 |
|  | 0.3319 |  | 8000 |
|  | 0.3203 |  | 8500 |
|  | 0.3066 |  | 9000 |
|  | 0.3024 | 0.3257 | 9179 |
|  |  | 0.3455 | 9500 |
|  |  | 0.3728 | 10000 |
|  |  | 0.3985 | 10500 |
|  |  | 0.4258 | 11000 |
|  |  | 0.4479 | 11500 |
|  |  | 0.4707 | 12000 |
|  |  | 0.4905 | 12500 |
|  |  | 0.5106 | 13000 |
|  |  | 0.5277 | 13500 |
|  |  | 0.5441 | 14000 |
|  |  | 0.5611 | 14500 |
|  |  | 0.5747 | 15000 |
|  |  | 0.5884 | 15500 |
|  |  | 0.6023 | 16000 |
|  |  | 0.6121 | 16500 |
|  |  | 0.6219 | 17000 |
|  |  | 0.6319 | 17500 |
|  |  | 0.6421 | 18000 |
|  |  | 0.6508 | 18500 |
|  |  | 0.6598 | 19000 |
|  |  | 0.6697 | 19500 |
|  |  | 0.6776 | 20000 |
|  |  | 0.6862 | 20500 |
|  |  | 0.6932 | 21000 |
|  |  | 0.6993 | 21500 |
|  |  | 0.7078 | 22000 |
|  |  | 0.7143 | 22500 |
|  |  | 0.7209 | 23000 |
|  |  | 0.7281 | 23500 |
|  |  | 0.7329 | 24000 |
|  |  | 0.7389 | 24500 |
|  |  | 0.7439 | 25000 |
|  |  | 0.7488 | 25500 |
|  |  | 0.7541 | 26000 |
|  |  | 0.7583 | 26500 |
|  |  | 0.7638 | 27000 |
|  |  | 0.7673 | 27500 |
|  |  | 0.7705 | 28000 |
|  |  | 0.7750 | 28500 |
|  |  | 0.7781 | 29000 |
|  |  | 0.7816 | 29500 |
|  |  | 0.7848 | 30000 |
|  |  | 0.7879 | 30500 |
|  |  | 0.7908 | 31000 |
|  |  | 0.7950 | 31500 |
|  |  | 0.7984 | 32000 |
|  |  | 0.8015 | 32500 |
|  |  | 0.8041 | 33000 |
|  |  | 0.8062 | 33500 |
|  |  | 0.8083 | 34000 |
|  |  | 0.8116 | 34500 |
|  |  | 0.8133 | 35000 |
|  |  | 0.8156 | 35500 |
|  |  | 0.8174 | 36000 |
|  |  | 0.8187 | 36500 |
|  |  | 0.8212 | 37000 |
|  |  | 0.8231 | 37500 |
|  |  | 0.8252 | 38000 |
|  |  | 0.8273 | 38500 |
|  |  | 0.8299 | 39000 |
|  |  | 0.8319 | 39500 |
|  |  | 0.8329 | 40000 |
|  |  | 0.8337 | 40500 |
|  |  | 0.8347 | 41000 |
|  |  | 0.8362 | 41500 |
|  |  | 0.8372 | 42000 |
|  |  | 0.8390 | 42500 |
|  |  | 0.8401 | 43000 |
|  |  | 0.8419 | 43500 |
|  |  | 0.8434 | 44000 |
|  |  | 0.8442 | 44500 |
|  |  | 0.8450 | 45000 |
|  |  | 0.8464 | 45500 |
|  |  | 0.8477 | 46000 |
|  |  | 0.8489 | 46500 |
|  |  | 0.8495 | 47000 |
|  |  | 0.8507 | 47500 |
|  |  | 0.8516 | 48000 |
|  |  | 0.8529 | 48500 |
|  |  | 0.8535 | 49000 |
|  |  | 0.8548 | 49500 |
|  |  | 0.8562 | 50000 |
