# Supplementary material for: The Cost Effectiveness of Psychological and Pharmacological Interventions for Social Anxiety Disorder: A Model-Based Economic Analysis
Source: PLoS One. 2015 Oct 27;10(10):e0140704. doi: 10.1371/journal.pone.0140704 (PMC4624770; doi:10.1371/journal.pone.0140704)
Supplement: S3 Table — (DOCX) [file pone.0140704.s006.docx]

**Ranking of interventions by cost effectiveness at different time horizons of the economic model – deterministic analysis**

| **Time horizon 1 year**  **+ 12 weeks** | **Time horizon 3 years**  **+ 12 weeks** | **Time horizon 5 years**  **+ 12 weeks**  **(base-case analysis)** | **Time horizon 10 years**  **+ 12 weeks** |
| --- | --- | --- | --- |
| Phenelzine | ICBT, C&W | ICBT, C&W | ICBT, C&W |
| SHNS, book | Phenelzine | ICBT, general | ICBT, general |
| Paroxetine | SHNS, book | Phenelzine | ICBT Hope |
| Sertraline | ICBT, general | SHNS, book | ICBT, short |
| Venlafaxine | SHWS, book | SHWS, book | Phenelzine |
| Escitalopram | Paroxetine | ICBT Hope | SHWS, internet |
| Fluoxetine | SHWS, internet | SHWS, internet | SHWS, book |
| SHWS, book | Venlafaxine | Paroxetine | GCBT, general |
| Fluvoxamine | Sertraline | GCBT, general | SHNS, book |
| Citalopram | Fluvoxamine | ICBT, short | Exposure |
| Mirtazapine | ICBT Hope | Exposure | GCBT, Heimberg |
| Moclobemide | Escitalopram | Venlafaxine | Paroxetine |
| SHWS, internet | GCBT, general | GCBT, Heimberg | SHNS, internet |
| Pregabalin | Fluoxetine | Sertraline | Venlafaxine |
| Placebo | Citalopram | Fluvoxamine | Fluvoxamine |
| SHNS, internet | Exposure | Escitalopram | PDPT |
| ICBT, C&W | Mirtazapine | Fluoxetine | Sertraline |
| Wait list | GCBT, Heimberg | SHNS, internet | Escitalopram |
| GCBT, general | SHNS, internet | Citalopram | Fluoxetine |
| ICBT, general | ICBT, short | Mirtazapine | Citalopram |
| Exposure | Moclobemide | Moclobemide | Mirtazapine |
| GCBT, Heimberg | Pregabalin | Pregabalin | Moclobemide |
| Mindfulness | Mindfulness | Mindfulness | Pregabalin |
| ICBT Hope | Placebo | Placebo | IPT |
| ICBT, short | Wait list | PDPT | Mindfulness |
| IPT | PDPT | Wait list | Supportive therapy |
| Supportive therapy | IPT | IPT | Placebo |
| PDPT | Supportive therapy | Supportive therapy | Wait list |

C&W: Clark and Wells model; GCBT: group cognitive behavioural therapy; ICBT: individually delivered cognitive behavioural therapy; IPT: interpersonal therapy; PDPT: psychodynamic psychotherapy; SHNS: self-help no support; SHWS: self-help with support
