# Supplementary material for: The Cost Effectiveness of Psychological and Pharmacological Interventions for Social Anxiety Disorder: A Model-Based Economic Analysis
Source: PLoS One. 2015 Oct 27;10(10):e0140704. doi: 10.1371/journal.pone.0140704 (PMC4624770; doi:10.1371/journal.pone.0140704)
Supplement: S4 Table — (DOCX) [file pone.0140704.s007.docx]

**Ranking of interventions by cost effectiveness at different values of the risk ratio of relapse of drugs versus psychological interventions (year 1) – deterministic analysis**

| **Risk ratio 1.00** | **Risk ratio 1.25** | **Risk ratio 1.50** | **Risk ratio 1.75** | **Risk ratio 2.00** | **Risk ratio 3.00**  **(base-case analysis)** |
| --- | --- | --- | --- | --- | --- |
| Phenelzine | ICBT, C&W | ICBT, C&W | ICBT, C&W | ICBT, C&W | ICBT, C&W |
| ICBT, C&W | Phenelzine | Phenelzine | Phenelzine | Phenelzine | ICBT, general |
| Paroxetine | ICBT, general | ICBT, general | ICBT, general | ICBT, general | Phenelzine |
| Venlafaxine | Paroxetine | SHNS, book | SHNS, book | SHNS, book | SHNS, book |
| Sertraline | SHNS, book | Paroxetine | SHWS, book | SHWS, book | SHWS, book |
| Fluvoxamine | Venlafaxine | SHWS, book | Paroxetine | Paroxetine | ICBT Hope |
| Escitalopram | Sertraline | Venlafaxine | SHWS, internet | SHWS, internet | SHWS, internet |
| Fluoxetine | Fluvoxamine | Sertraline | Venlafaxine | ICBT Hope | Paroxetine |
| ICBT, general | SHWS, book | Fluvoxamine | ICBT Hope | Venlafaxine | GCBT, general |
| SHNS, book | Escitalopram | SHWS, internet | Sertraline | Sertraline | ICBT, short |
| Citalopram | Fluoxetine | ICBT Hope | Fluvoxamine | Fluvoxamine | Exposure |
| Mirtazapine | Citalopram | Escitalopram | Escitalopram | GCBT, general | Venlafaxine |
| SHWS, book | SHWS, internet | Fluoxetine | GCBT, general | ICBT, short | GCBT, Heimberg |
| SHWS, internet | Mirtazapine | Citalopram | Fluoxetine | Exposure | Sertraline |
| Moclobemide | ICBT Hope | GCBT, general | ICBT, short | Escitalopram | Fluvoxamine |
| ICBT, Hope | GCBT, general | Mirtazapine | Exposure | Fluoxetine | Escitalopram |
| Pregabalin | Exposure | Exposure | Citalopram | GCBT, Heimberg | Fluoxetine |
| GCBT, general | Moclobemide | ICBT, short | Mirtazapine | Citalopram | SHNS, internet |
| Exposure | ICBT, short | GCBT, Heimberg | GCBT, Heimberg | Mirtazapine | Citalopram |
| GCBT, Heimberg | GCBT, Heimberg | SHNS, internet | SHNS, internet | SHNS, internet | Mirtazapine |
| ICBT, short | Pregabalin | Moclobemide | Moclobemide | Moclobemide | Moclobemide |
| SHNS, internet | SHNS, internet | Pregabalin | Pregabalin | Pregabalin | Pregabalin |
| Pill placebo | Placebo | Placebo | Mindfulness | Mindfulness | Mindfulness |
| Wait list | Mindfulness | Mindfulness | Placebo | Placebo | Placebo |
| Mindfulness | Wait list | Wait list | Wait list | Wait list | PDPT |
| PDPT | PDPT | PDPT | PDPT | PDPT | Wait list |
| IPT | IPT | IPT | IPT | IPT | IPT |
| Supportive therapy | Supportive therapy | Supportive therapy | Supportive therapy | Supportive therapy | Supportive therapy |

C&W: Clark and Wells model; GCBT: group cognitive behavioural therapy; ICBT: individually delivered cognitive behavioural therapy; IPT: interpersonal therapy; PDPT: psychodynamic psychotherapy; SHNS: self-help no support; SHWS: self-help with support.
